# Supplementary material for: Cognitive decline in patients with prostate cancer: study protocol of a prospective cohort, NEON-PC
Source: BMJ Open. 2021 Feb 15;11(2):e043844. doi: 10.1136/bmjopen-2020-043844 (PMC7887367; doi:10.1136/bmjopen-2020-043844)
Supplement: Author's manuscript [file bmjopen-2020-043844.draft_revisions.pdf]

BMJ Open

BMJ Open is committed to open peer review. As part of this commitment we make the peer review history of every article we publish publicly available.

When an article is published we post the peer reviewers' comments and the authors' responses online. We also post the versions of the paper that were used during peer review. These are the versions that the peer review comments apply to.

The versions of the paper that follow are the versions that were submitted during the peer review process. They are not the versions of record or the final published versions. They should not be cited or distributed as the published version of this manuscript.

BMJ Open is an open access journal and the full, final, typeset and author-corrected version of record of the manuscript is available on our site with no access controls, subscription charges or pay-per-view fees (http://bmjopen.bmj.com).

If you have any questions on BMJ Open's open peer review process please email info.bmjopen@bmj.com

Neuro-oncological complications of prostate cancer: study protocol of a prospective cohort study on cognitive decline

Journal:	*BMJ Open*
Manuscript ID	bmjopen-2020-043844
Article Type:	Protocol
Date Submitted by the Author:	14-Aug-2020

Complete List of Authors:	Araujo, Natalia; University of Porto Epidemiology Research Unit<br>Morais, Samantha; University of Porto Epidemiology Research Unit; University of Porto Faculty of Medicine, Department of Clinical Epidemiology, Predictive Medicine and Public Health<br>Costa, Ana ; University of Porto Epidemiology Research Unit<br>Braga, Raquel; University of Porto Epidemiology Research Unit; Unidade Local de Saúde de Matosinhos EPE<br>Carneiro, Ana; Francisco Gentil Portuguese Institute for Oncology of Porto, Oncology<br>Cruz, Vitor; University of Porto Epidemiology Research Unit; Unidade Local de Saúde de Matosinhos EPE, Neurology<br>Ruano, Luis; University of Porto Epidemiology Research Unit; Centro Hospitalar de Entre o Douro e Vouga EPE, Neurology<br>Oliveira, Jorge; Francisco Gentil Portuguese Institute for Oncology of Porto, Urology<br>Figueiredo, Luis; Universidade do Minho Instituto de Investigação em Ciências da Vida e Saúde, Medicine School; Centro Hospitalar de São João EPE, Urology<br>Pereira, Susana; University of Porto Epidemiology Research Unit; Francisco Gentil Portuguese Institute for Oncology of Porto, Neurology<br>Lunet, Nuno; University of Porto Faculty of Medicine, Department of Clinical Epidemiology, Predictive Medicine and Public Health; University of Porto Epidemiology Research Unit

Keywords:	Neurological oncology < NEUROLOGY, Epidemiology < ONCOLOGY, Urological tumours < ONCOLOGY, Delirium & cognitive disorders < PSYCHIATRY

SCHOLARONE™ Manuscripts

I, the Submitting Author has the right to grant and does grant on behalf of all authors of the Work (as defined in the below author licence), an exclusive licence and/or a non-exclusive licence for contributions from authors who are: i) UK Crown employees; ii) where BMJ has agreed a CC-BY licence shall apply, and/or iii) in accordance with the terms applicable for US Federal Government officers or employees acting as part of their official duties; on a worldwide, perpetual, irrevocable, royalty-free basis to BMJ Publishing Group Ltd ("BMJ") its licensees and where the relevant Journal is co-owned by BMJ to the co-owners of the Journal, to publish the Work in this journal and any other BMJ products and to exploit all rights, as set out in our licence.

The Submitting Author accepts and understands that any supply made under these terms is made by BMJ to the Submitting Author unless you are acting as an employee on behalf of your employer or a postgraduate student of an affiliated institution which is paying any applicable article publishing charge ("APC") for Open Access articles. Where the Submitting Author wishes to make the Work available on an Open Access basis (and intends to pay the relevant APC), the terms of reuse of such Open Access shall be governed by a Creative Commons licence – details of these licences and which Creative Commons licence will apply to this Work are set out in our licence referred to above.

Other than as permitted in any relevant BMJ Author's Self Archiving Policies, I confirm this Work has not been accepted for publication elsewhere, is not being considered for publication elsewhere and does not duplicate material already published. I confirm all authors consent to publication of this Work and authorise the granting of this licence.

For peer review only

Neuro-oncological complications of prostate cancer: study protocol of a prospective cohort study on cognitive decline

Natália Araújo[1], Samantha Morais[1,2], Ana Rute Costa[1], Raquel Braga[1,3], Filipa Carneiro[4], Vítor Tedim Cruz[1,5], Luís Ruano[1,6], Jorge Oliveira[4], Luís Pacheco-Figueiredo[7,8], Susana Pereira[1,4], Nuno Lunet[1,2]

[1] EPIUnit – Instituto de Saúde Pública, Universidade do Porto, Porto, Portugal

[2] Departamento de Ciências da Saúde Pública e Forenses e Educação Médica, Faculdade de Medicina da Universidade do Porto, Porto, Portugal

[3] Unidade Local de Saúde de Matosinhos, Matosinhos, Portugal

[4] Instituto Português de Oncologia do Porto, Porto, Portugal

[5] Serviço de Neurologia, Unidade de Saúde Local de Matosinhos, Matosinhos, Portugal

[6] Serviço de Neurologia, Centro Hospitalar Entre Douro e Vouga, Santa Maria da Feira, Portugal

[7] Instituto de Investigação em Ciências da Vida e Saúde, Escola de Medicina da Universidade do Minho, Braga, Portugal

[8] Serviço de Urologia, Centro Hospitalar São João, Porto, Portugal

Corresponding author:

Nuno Lunet – nlunet@med.up.pt

Departamento de Ciências da Saúde Pública e Forenses e Educação Médica, Faculdade de Medicina da Universidade do Porto

Al. Prof. Hernâni Monteiro

4200-319 Porto, Portugal

Phone: +351 225 513 652; Fax: +351 225 513 653

For peer review only

Abstract

Introduction: Prostate cancer is the most prevalent oncologic disease among men in industrialized countries. Despite the high survival rates, treatments are often associated with adverse effects, including metabolic and cardiovascular complications, sexual dysfunction and, to a lesser extent, cognitive decline. This study was primarily designed to evaluate the trajectories of cognitive performance in prostate cancer patients, and to quantify the impact of the disease and its treatments on the occurrence of cognitive decline.

Methods: Participants will be recruited from two main hospitals providing care to approximately half the prostate cancer patients in Northern Portugal (Portuguese Institute of Oncology of Porto and São João Hospital Centre), and will comprise a cohort of recently diagnosed prostate cancer patients proposed for different treatment plans, including: 1) radical prostatectomy; 2) brachytherapy and/or radiotherapy; 3) radiotherapy in combination with androgen deprivation therapy; 4) androgen deprivation therapy (with or without chemotherapy). Recruitment began in February 2018 and is expected to continue until the first semester of 2021. Follow-up evaluations will be conducted at one, three, five, seven and 10 years. Sociodemographic, behavioral and clinical characteristics, anxiety and depression, health literacy, health status, quality of life, and sleep quality will be assessed. Blood pressure and anthropometrics will be measured, and a fasting blood sample will be collected. Participants' cognitive performance will be evaluated before treatments and throughout follow-up (Montreal Cognitive Assessment and Cube Test as well as Brain on Track for remote monitoring). All participants suspected of cognitive impairment will undergo neuropsychological tests and clinical observation by a neurologist.

Ethics and dissemination: The study was approved by the Ethics Committee of the hospitals involved. All participants will provide written informed consent, and study procedures will be developed to ensure data protection and confidentiality. Results will be disseminated through publication in peer-reviewed journals and presentation in scientific meetings.

For peer review only

Article Summary

Strengths and limitations of this study

- This protocol describes a prospective cohort study of prostate cancer patients, expected to reflect the contemporary patterns of diagnosis and treatment in developed countries.
- Cognitive impairment will be characterized regarding its severity and possible aetiologies through neuropsychological and clinical evaluations.
- Short- and long-term effects as well as mediators of the effect of androgen deprivation therapy on cognitive performance will be analysed.
- A longitudinal remote monitoring tool of cognitive function will be used, in addition to state-of-the-art methods, which allows for more frequent standardized evaluations, while reducing learning effects of repeated measurements.

For peer review only

-

Introduction

Prostate cancer is the second most common neoplasm and the fifth leading cause of death from cancer among men, with an estimated 1.3 million new cases and 359 thousand deaths in 2018 worldwide.[1] In recent decades, prostate cancer incidence has been heavily influenced by diagnoses following prostate-specific antigen testing of asymptomatic individuals and by the detection of latent cancer in tissue removed during prostatectomy or autopsy.[1] At the same time, prostate cancer mortality has been decreasing in many countries, which has been linked to earlier diagnosis because of extensive use of prostate-specific antigen screening, and improved treatment including radical prostatectomy, hormonal therapy and radiation therapy.[2 3] Increases in prostate cancer survival[4] require a comprehensive assessment of the burden of cancer, due to the disease, treatment and sequelae.[5 6]

Androgen deprivation therapy is used in the treatment of approximately half of all prostate cancer patients,[7 8] and it may last from six to 36 months, on an intermittent basis or continue indefinitely.[9] The use of androgen deprivation therapy and its impact on cognitive function has been assessed, both in prospective studies evaluating cognitive performance using neuropsychological tests and in large retrospective studies reporting the risk of dementia or of Alzheimer's disease in prostate cancer patients according to androgen deprivation therapy exposure.[10-12] However, methodological heterogeneity does not allow for the direct comparison of results, and shortcomings of the study designs, including small sample sizes, short follow-up periods or limited quality of information on cognitive status, as well as residual confounding, preclude more robust conclusions on this topic.[10] Also, in addition to the possible direct effect of androgen deprivation therapy on cognitive function due to the drop in serum testosterone and its biological activity in certain areas of the brain,[13] hormonal changes may also cause metabolic alterations,[14] with an increase in cardiovascular risk factors, such as an increase in insulin resistance, serum cholesterol and triglycerides, or anemia,[15] which in turn are associated to cognitive decline.[16-19] This possible indirect effect may take longer to manifest at the brain than the direct decrease in testosterone serum levels, and it may be related to the development of dementia. The potential mediator effect of these biochemical and hematologic parameters has not been studied. Prospective investigations

For peer review only

including an accurate characterization of the cognitive performance of prostate cancer patients proposed for different types of treatment, and analyses accounting for distinct causal pathways may contribute to a better understanding of the effects of prostate cancer and its treatments on cognitive decline.

Therefore, this project primarily aims to understand the impact of androgen deprivation therapy on the cognitive performance of prostate cancer patients in Northern Portugal. The main specific objectives are as follows:

1) To describe the trajectories of cognitive performance over time (up to 10 years) in prostate cancer patients under different treatments and, in comparison to the general population, by using the Montreal Cognitive Assessment tool, the Cube Test and Brain on Track®. The relation between patients' characteristics, cancer treatments and different cognitive trajectories will also be assessed.

2) To quantify the association between androgen deprivation therapy and cognitive decline, in the short- and in the long-term.

3) To assess the role of metabolic syndrome and anemia as possible mediators of the androgen deprivation therapy effect on cognitive performance.

Methods and analysis

We describe a prospective cohort study that will evaluate prostate cancer patients selected among those being treated at the two largest hospitals providing cancer care in the North of Portugal, which attend half the prostate cancer patients in this region.

Eligibility criteria

Eligible participants are those with a recent diagnosis of prostate cancer and being initially proposed for radical prostatectomy (group 1), brachytherapy or radiotherapy (group 2), radiotherapy in combination with androgen deprivation therapy (group 3), or androgen deprivation therapy with or without chemotherapy (group 4), and prostate cancer survivors never treated with androgen deprivation therapy

For peer review only

before, who present with a recurrence of the disease to be treated with androgen deprivation therapy, with or without chemotherapy (group 5).

Participants who had a previous chemotherapy or radiotherapy treatment for another primary cancer, or a diagnosis of a psychiatric or a neurologic condition impairing cognitive function before the prostate cancer diagnosis, or being unable to understand the purpose of the study or to collaborate will be excluded, as well as those expected to receive cancer treatments outside the Portuguese Institute of Oncology of Porto or the São João Hospital Centre.

Participants' recruitment and follow-up

Prostate cancer patients will be consecutively recruited at the Portuguese Institute of Oncology of Porto and the São João Hospital Centre, from February 2018 to the first semester of 2021. Participants will be evaluated at baseline (before any treatment for recently diagnosed patients or before androgen deprivation therapy for patients with a recurrence of the disease), and at one, three, five, seven and 10 years after enrolment, as depicted in Figure 1.

Data collected from medical records

Clinical characteristics, including co-morbidities, medication and cancer treatment, as well as prognostic and treatment response biomarkers will be systematically collected by medical doctors from the patients´ medical records.

Questionnaire evaluation

Data on sociodemographic (birth date, address, marital status, education, occupation), lifestyle and dietary characteristics (smoking and alcohol consumption, and intake of fruits and vegetables, physical activity and sedentary behaviours) will be collected through questionnaires applied by a trained interviewer. Anxiety and depression,[20 21] sleep quality,[22 23] quality of life and health status,[24-27] and health literacy[28 29] will be evaluated through self-administered questionnaires validated for the Portuguese population, and are described in detail in Table 1.

For peer review only

Blood pressure

Blood pressure will be measured with a digital blood pressure monitor (Omron M6). Participants will be asked to remain seated, with the right arm and back supported and feet firmly on the floor, and to abstain from speaking during the entire procedure. The cuff will be placed on the right arm so the bottom margin is approximately two to three centimeters above the antecubital fossa. A larger or a smaller cuff will be used as necessary to fit the arm of the participant. Three measurements with one-minute intervals will be registered.

Anthropometrics

Weight and height will be measured with participants in light clothes and no shoes, and registered to the nearest kilogram and centimeter, respectively, using a digital column scale (Seca 799). Waist and hip perimeters will be measured using a non-elastic measuring tape (Seca 211) with participants standing, with feet slightly apart and the arms relaxed along the body; waist perimeter will be measured at half the distance between the last rib and the iliac crest. Hip perimeter will be measured with participants in the same position, with the measuring tape placed at the widest part of the hip below the iliac crest. Both waist and hip circumferences will be registered to the nearest millimeter. Most measurements are expected to be performed in the morning.

Blood sample

A fasting blood sample (at least 12 hours) will be collected by the hospitals´ nurses using venous puncture, and blood samples will be centrifuged at 3000 rpm for 10 minutes to obtain plasma and serum, within 30 to 60 minutes. Total cholesterol, high density lipoprotein cholesterol, triglycerides, glycaemia, glycated hemoglobin and hemoglobin will be analysed. Plasma and serum samples will be stored in small aliquots at -80º C until the end of the study (10 years).

For peer review only

Cognitive function evaluation

Cognitive function will be evaluated using the Montreal Cognitive Assessment[30 31] and the Cube Test,[32] at baseline and at each of the subsequent follow-up evaluations, and with a web-based tool for remote longitudinal assessment (Brain on Track),[33] every three months for a period of up to 10 years.

Participants suspected of cognitive impairment will undergo a comprehensive neuropsychological assessment that will allow specific domains of cognitive function to be analysed; the battery of tests is described in Table 2. Additionally, those with confirmed cognitive impairment will undergo a clinical evaluation by a neurologist.

The Montreal Cognitive Assessment

The Montreal Cognitive Assessment is a pen and paper screening test, developed to detect mild cognitive impairment. It assesses eight cognitive domains (visuospatial ability, executive function, attention, concentration, working memory, language, verbal memory and orientation), generating a total score ranging from 0 to 30.[30] The translated, culturally adapted and validated version of the Montreal Cognitive Assessment for the Portuguese population[34] will be used, and the performance of participants will be classified as probable cognitive impairment when the score is 1.5 standard deviations below the mean of age- and education-based group distribution from published normative data.[31]

The Cube Test

The Cube Test will be used as a rapid cognitive screening tool, which can be applied to illiterate participants, or those with low educational levels, language or hearing deficits.[32] The Cube Test is easy to apply, and the simple instructions and scoring procedures contribute for standardized use. The test is based on the time spent in assembling the six faces of a 3D cube and then, the correct placement of the six faces of the 3D cube on a grid with 25 squares measuring eight by eight centimetres. The Cube Test assesses visuoconstructive, visuospatial and executive functions, visuospatial working memory, information processing speed, incidental learning, motor processing speed, and manual dexterity.

For peer review only

Brain on Track

The Brain on Track test will be used for the remote evaluation of changes in cognitive function over a two-year period. This is a computerised cognitive monitoring test, which was developed and validated in the Portuguese population, showing good internal consistency, discriminative ability and reliability.[33 35] Brain on Track evaluates different cognitive domains, including attention, memory, executive functions, language, calculation, constructive capacity and visuospatial processing, through 11 exercises designed to include random elements and alternate sequences to lower the learning effect of repeating cognitive tests. It is to be performed using a home computer to access a web platform where different cognitive tests are uploaded. Each patient´s results are stored and can be monitored by the research team.

Patients will be eligible to participate if they have completed at least three years of schooling, have no severe motor, visual or language impairments that prevent cognitive assessment, have easy access to a computer with an internet connection, and are able to use a computer without help. At the end of the baseline evaluation, participants will undergo a training session, and will be instructed to remotely log into the web platform and proceed to the first evaluation after one week, and then every three months for a period of two years. A Short Message Service reminder will be sent to participants one day before each remote evaluation. The research team will be automatically notified when participants fail to perform the test in order to re-schedule the evaluation.

Comprehensive neuropsychological assessment and clinical evaluation by a neurologist

All participants who score below 1.5 standard deviations of age- and education-adjusted cut-offs on the Montreal Cognitive Assessment[30 31] in each evaluation (baseline, and one, three, five, seven and 10 years of follow-up) will undergo a neuropsychological evaluation, expectedly within one-month, comprising a battery of cognitive tests (Table 2). The type and progressive nature of cognitive impairment, and its functional impact will be determined through a clinical evaluation performed by a neurologist, with a close surrogate present. Additionally, participants with a first neuropsychological evaluation will be re-assessed with the same battery of tests, independently of their Montreal Cognitive Assessment score in the subsequent follow-up evaluations. A random sample of 30 patients with normal MoCA scores will also

perform a neuropsychological evaluation at one, three, five, seven and ten years of follow-up, as a control group.

Participants will be classified as having mild cognitive impairment when presenting cognitive complaints over a period of at least six months, as reported by the patient or family members, modest cognitive decline from a previous level of performance reported by the patient or family members, and neuropsychological evaluation scores at least 2.0 standard deviations below the age-corrected norms in at least one cognitive domain or at least 1.5 standard deviations below the age-corrected norms in at least two cognitive domains, while also presenting no clinical depression or interference of cognitive function with independence in daily activities.[36 37]

Dementia will be defined according to the criteria used for defining major neurocognitive disorder of the *Diagnostic and Statistical Manual of Mental Disorders* (fifth edition), that is significant cognitive impairment in at least one cognitive domain representing a significant decline from a previous level of functioning that interferes with independence in daily activities.[37] The severity of dementia will be classified using the clinical dementia rating scale.[38] The initial clinical classification will be confirmed after at least six months of clinical follow-up by a neurologist, and a complete diagnostic workup to identify other potential causes of cognitive impairment not related to oncologic disease, including blood analyses for treatable causes of dementia and imaging studies.

Data analyses and sample size

Trajectories of cognitive decline will be described through indicators of cognitive performance at different moments of evaluation using the appropriate format according to the nature of the variables and their distribution. Fixed- and mixed-effects models will be computed to compare cognitive performance trajectories (considering age and education) over time, according to other sociodemographic and clinical characteristics, for each of the treatment groups.

Prevalence at baseline and incidence measures (incidence rates and cumulative incidences) and the corresponding 95% confidence intervals will be estimated to quantify the frequency of cognitive impairment, and the association between treatments and incident cognitive impairment. Cumulative

For peer review only - http://bmjopen.bmj.com/site/about/guidelines.xhtml

incidence will be estimated considering death as a competitive event, according to the Kalbfleisch and Prentice method.[39] Crude and adjusted relative risks will be calculated.

The sample size was calculated considering the objective to quantify the association between the use of androgen deprivation therapy and cognitive decline assessed with the Montreal Cognitive Assessment, between the baseline and the one-year evaluation. For this, assuming a statistical power of 80%, a level of significance of 5% and a 1:1 ratio between androgen deprivation therapy-exposed and unexposed, 600 prostate cancer patients will be necessary to detect a two-fold higher proportion of participants (14%) with a cognitive change below 1.5 standard deviations of the mean of change in the androgen deprivation therapy group.

For the description of cognitive performance trajectories, and the calculation of the prevalence of cognitive impairment at baseline and incidence measures (incidence rates and cumulative incidences), the sample size will influence the precision of the estimates at each moment but will not be a limiting factor for the essentially descriptive accomplishment of these objectives. Nevertheless, considering the prevalence of cognitive impairment in the general population of Northern Portugal of 9.6%,[40] a precision of 2.4%, and a 95% confidence level, a sample of 579 individuals will be needed. As such, the estimated sample size calculated above will also be sufficient for estimating the prevalence of cognitive impairment in the population of prostate cancer patients.

Considering the high potential for confounding by indication, propensity scores calculated based on several disease characteristics, including prognostic biomarkers and predictors of response to treatment, will be used to adjust the models.

Training of interviewers and the use of standardized procedures for data collection are expected to contribute to a low proportion of missing data, and no imputation is being planned.

Considering our experience in another cancer cohort,[41] we estimate that approximately a third of the total sample will participate in the Brain on Track evaluation. Using as criteria for referral of participants to the comprehensive neuropsychological assessment, the Montreal Cognitive Assessment cut-off score, 1.5 standard deviations below the mean of age and education-based group distribution from published

For peer review only

normative data[31], we expect at least 42 patients to undergo a neuropsychological assessment at baseline and at each subsequent evaluation.

Taking into account the survival of prostate cancer patients in the North of Portugal,[42] and the high participation obtained in a previous prospective cohort study of breast cancer patients;[41 43] we estimate that at least 90% and 80% of patients will participate in the one- and five-year follow-up evaluations, respectively. In order to minimize refusals and losses to follow-up, all evaluations will be scheduled to take place on the same day as routine appointments in the respective hospital and participants will be invited again when they miss scheduled appointments.

Contingency plan

Due to the COVID-19 pandemic, recruitment and evaluation of participants were interrupted from March to June 2020. Beginning in July, procedures were adapted to minimize the risk of infection for participants and members of the research team. Only the Montreal Cognitive Assessment will be performed face-to-face at the hospital. Participants will answer the questionnaire on sociodemographic, and lifestyle and dietary characteristics during a telephone interview. Self-administered questionnaires will be completed at home and sent mailed back with a pre-paid envelope.

Anthropometrics measurements, blood sample collection and the Cube test evaluation will not be performed. Weight, height, blood pressure and blood sample parameters will be retrieved from medical records when available or asked to the participants. The initial training session for the Brain on Track evaluation will be conducted through videoconference.

Patient and Public Involvment

Patients and public were not involved in the conception, design, and dissemination of this study.

Ethics and dissemination

For peer review only

Ethics approval was obtained from the Ethics Committees of the Portuguese Institute of Oncology of Porto (Ref. CES 89/017) and the São João Hospital Centre (Ref. 76/17) and by the Portuguese Data Protection Authority (Authorization 3478/2017). Written informed consent will be obtained from all participants after the project´s aims and procedures are fully explained by a member of the research team.

This is an observational study in which prostate cancer patients will be followed according to usual clinical practice, as such the occurrence of harmful effects related to participation in the study are not expected. Participants will receive detailed information about the research purpose and objectives, name and institution of the researchers, expected duration of the interview, voluntary nature of participation, clearly stating that there will be no penalty for those who refuse to participate, and ensuring confidentiality and anonymity of all the information provided. Participants will be asked to give authorization for collection of data from their personal clinical records. After clarification of any doubts, an informed consent will be signed in duplicate and a copy will be given to each participant. All participants will be informed that they can leave the study at any time, and this decision does not affect their medical care. There is no expected risk or discomfort other than those arising from interviewing, collecting venous blood samples and physical measurements (height, weight, blood pressure). Only participants able to understand the study and provide informed consent will be included. To minimize possible discomfort due to the required trips to the hospital for face-to-face evaluations or the duration of interviews, and to avoid unnecessary burden and travel expenses, data collection procedures were designed to last no more than 60 minutes, and will be scheduled to take place on the same day as other appointments in the respective hospitals as part of regular clinical care, preferably in the morning due to the fasting requirement. Starting in July 2020, only the Montreal Cognitive Assessment will be performed face-to-face at the hospital, to reduce the risk of infection by the severe acute respiratory syndrome coronavirus 2 (SARS-CoV-2).

This study requires the collection and processing of sensitive personal data including health and clinical data from questionnaires and the clinical files of patients. Therefore, additional measures will be taken to protect the anonymity and the confidentiality of all participants. All data regarding clinical aspects will be collected by clinical members of the research team and privacy is assured. All participants will have a study-specific identification number, which will be used in all questionnaires and stored blood samples.

For peer review only

The correspondence between this identification number and the personal identifiable information will be stored in a file, to which only the principal investigator will have access. Only the research team will have access to the database with anonymized data, saved on a password-protected secure computer. No personal identifiers will be used in data analyses. The same procedures will be adopted for each of the evaluations.

The expected results may contribute to elucidate the magnitude of the androgen deprivation therapy effect on the cognitive function of prostate cancer patients, and the possible mediator effect of metabolic syndrome and anemia in this process. This may help clinical decisions regarding the pharmacological class to be used in patients more vulnerable to cognitive impairment. This study may also contribute to the refinement and validation of the longitudinal monitoring tool Brain on Track. Considering the 10-year temporal horizon of this project, the follow-up of the cohort assembled will contribute to a better understanding of the long-term trajectories of cognitive performance and the iatrogenic effects of prostate cancer treatments.

The findings of this project will be submitted for publication in international peer-reviewed journals, and proposed for presentation in relevant national and international conferences, which will allow for the dissemination of the main findings across the medical community. Press releases through mass media will also be issued to promote the dissemination of information relevant to the general population and policy makers. Furthermore, the project will contribute to the training of researchers through the production of masters´ theses and doctoral dissertations.

For peer review only

Authors´ contributions

NL and SP conceived and designed the study. NA wrote the first version of the manuscript. ARC, FC, JO, LPF, LR, NL, RB, SM, SP and VTC critically revised the manuscript for relevant intellectual content. All authors approved the final version for submission.

Funding statement

This study was funded by FEDER through the Operational Programme Competitiveness and Internationalization and national funding from the Foundation for Science and Technology – FCT (Portuguese Ministry of Science, Technology and Higher Education) under the project "NEON-PC - Neuro-oncological complications of prostate cancer: longitudinal study of cognitive decline" (POCI-01-0145-FEDER-032358; Ref. PTDC/SAU-EPI/32358/2017), and the Unidade de Investigação em Epidemiologia - Instituto de Saúde Pública da Universidade do Porto (EPIUnit) (UIDB/04750/2020) financed by national funds from FCT. SM was funded under the scope of the project "NEON-PC - Neuro-oncological complications of prostate cancer: longitudinal study of cognitive decline" (POCI-01-0145-FEDER-032358; Ref. PTDC/SAU-EPI/32358/2017). Individual PhD grants attributed to ARC (SFRH/BD/102181/2014) and NA (SFRH/BD/119390/2016) were funded by FCT and the "Programa Operacional Capital Humano" (POCH/FSE).

Competing interests statement

All the authors declare having no competing interests.

For peer review only

References

1. Bray F, Ferlay J, Soerjomataram I, et al. Global cancer statistics 2018: GLOBOCAN estimates of incidence and mortality worldwide for 36 cancers in 185 countries. *CA: a cancer journal for clinicians* 2018 doi: 10.3322/caac.21492 [published Online First: 2018/09/13]

2. Fontes F, Severo M, Castro C, et al. Model-based patterns in prostate cancer mortality worldwide. *British journal of cancer* 2013;108(11):2354-66. doi: 10.1038/bjc.2013.217 [published Online First: 2013/05/11]

3. Wong MC, Goggins WB, Wang HH, et al. Global incidence and mortality for prostate cancer: analysis of temporal patterns and trends in 36 countries. *European urology* 2016;70(5):862-74. doi: 10.1016/j.eururo.2016.05.043 [published Online First: 2016/06/13]

4. Trama A, Foschi R, Larranaga N, et al. Survival of male genital cancers (prostate, testis and penis) in Europe 1999-2007: results from the EUROCARE-5 study. *European journal of cancer (Oxford, England : 1990)* 2015;51(15):2206-16. doi: 10.1016/j.ejca.2015.07.027 [published Online First: 2015/10/01]

5. Treanor CJ, Li J, Donnelly M. Cognitive impairment among prostate cancer patients: an overview of reviews. *European journal of cancer care* 2017;26(6) doi: 10.1111/ecc.12642 [published Online First: 2017/02/09]

6. McGinty HL, Phillips KM, Jim HS, et al. Cognitive functioning in men receiving androgen deprivation therapy for prostate cancer: a systematic review and meta-analysis. *Supportive care in cancer : official journal of the Multinational Association of Supportive Care in Cancer* 2014;22(8):2271-80. doi: 10.1007/s00520-014-2285-1 [published Online First: 2014/05/27]

7. Shahinian VB, Kuo YF, Freeman JL, et al. Increasing use of gonadotropin-releasing hormone agonists for the treatment of localized prostate carcinoma. *Cancer* 2005;103(8):1615-24. doi: 10.1002/cncr.20955 [published Online First: 2005/03/03]

8. Barry MJ, Fowler FJ, Jr., O'Leary M P, et al. The American Urological Association Symptom Index for Benign Prostatic Hyperplasia. *The Journal of urology* 2017;197(2S):S189-S97. doi: 10.1016/j.juro.2016.10.071 [published Online First: 2016/12/26]

9. National Comprehensive Cancer Network. NCCN Clinical Practice Guidelines in Oncology (NCCN Guideline). Prostate Cancer., 2020.

For peer review only

10. Kluger J, Roy A, Chao HH. Androgen Deprivation Therapy and Cognitive Function in Prostate Cancer. *Current oncology reports* 2020;22(3):24. doi: 10.1007/s11912-020-0884-1 [published Online First: 2020/02/13]

11. Sun M, Cole AP, Hanna N, et al. Cognitive Impairment in Men with Prostate Cancer Treated with Androgen Deprivation Therapy: A Systematic Review and Meta-Analysis. *Journal of Urology* 2018;199(6):1417-25. doi: doi:10.1016/j.juro.2017.11.136

12. McHugh DJ, Root JC, Nelson CJ, et al. Androgen-deprivation therapy, dementia, and cognitive dysfunction in men with prostate cancer: How much smoke and how much fire? *Cancer* 2018;124(7):1326-34.

13. Höfer P, Lanzenberger R, Kasper S. Testosterone in the brain: Neuroimaging findings and the potential role for neuropsychopharmacology. *European Neuropsychopharmacology* 2013;23(2):79-88. doi: https://doi.org/10.1016/j.euroneuro.2012.04.013

14. Bosco C, Crawley D, Adolfsson J, et al. Quantifying the evidence for the risk of metabolic syndrome and its components following androgen deprivation therapy for prostate cancer: a meta-analysis. *PloS one* 2015;10(3):e0117344. doi: 10.1371/journal.pone.0117344 [published Online First: 2015/03/21]

15. Timilshina N, Hussain S, Breunis H, et al. Predictors of hemoglobin decline in non-metastatic prostate cancer patients on androgen deprivation therapy: a matched cohort study. *Supportive care in cancer : official journal of the Multinational Association of Supportive Care in Cancer* 2011;19(11):1815-21. doi: 10.1007/s00520-010-1023-6 [published Online First: 2010/10/19]

16. Kim B, Feldman EL. Insulin resistance as a key link for the increased risk of cognitive impairment in the metabolic syndrome. *Experimental & molecular medicine* 2015;47:e149. doi: 10.1038/emm.2015.3 [published Online First: 2015/03/15]

17. Yaffe K. Metabolic syndrome and cognitive decline. *Current Alzheimer research* 2007;4(2):123-6. [published Online First: 2007/04/14]

18. Trevisan C, Veronese N, Bolzetta F, et al. Low hemoglobin levels and the onset of cognitive impairment in older people: the PRO.V.A. Study. *Rejuvenation research* 2016;19(6):447-55. doi: 10.1089/rej.2015.1768 [published Online First: 2016/01/19]

19. Faux NG, Rembach A, Wiley J, et al. An anemia of Alzheimer's disease. *Molecular psychiatry* 2014;19(11):1227-34. doi: 10.1038/mp.2013.178 [published Online First: 2014/01/15]

For peer review only

20. Zigmond AS, Snaith RP. The Hospital Anxiety and Depression Scale. *Acta psychiatrica Scandinavica* 1983;67(6):361-70. [published Online First: 1983/06/01]

21. Pais-Ribeiro J, Silva I, Ferreira T, et al. Validation study of a Portuguese version of the Hospital Anxiety and Depression Scale. *Psychology, health & medicine* 2007;12(2):225-35; quiz 35-7. doi: 10.1080/13548500500524088 [published Online First: 2007/03/17]

22. Buysse DJ, Reynolds CF, 3rd, Monk TH, et al. The Pittsburgh Sleep Quality Index: a new instrument for psychiatric practice and research. *Psychiatry research* 1989;28(2):193-213. doi: 10.1016/0165-1781(89)90047-4 [published Online First: 1989/05/01]

23. Marques D, Gomes AA, Meiavia A, et al. Reliability and initial validation of the Pittsburgh Sleep Quality Index, European Portuguese version: a preliminary study in a sample of higher education students. *Sleep Medicine* 2013;14:e140. doi: https://doi.org/10.1016/j.sleep.2013.11.316

24. Aaronson NK, Ahmedzai S, Bergman B, et al. The European Organization for Research and Treatment of Cancer QLQ-C30: a quality-of-life instrument for use in international clinical trials in oncology. *Journal of the National Cancer Institute* 1993;85(5):365-76. doi: 10.1093/jnci/85.5.365 [published Online First: 1993/03/03]

25. Pais-Ribeiro J, Pinto C, Santos C. Validation study of the Portuguese version of the QLC-C30-V.3. *Psicologia, Saúde & Doenças* 2008;9:89-102.

26. van Andel G, Bottomley A, Fossa SD, et al. An international field study of the EORTC QLQ-PR25: a questionnaire for assessing the health-related quality of life of patients with prostate cancer. *European journal of cancer (Oxford, England : 1990)* 2008;44(16):2418-24. doi: 10.1016/j.ejca.2008.07.030 [published Online First: 2008/09/09]

27. Herdman M, Gudex C, Lloyd A, et al. Development and preliminary testing of the new five-level version of EQ-5D (EQ-5D-5L). *Quality of life research : an international journal of quality of life aspects of treatment, care and rehabilitation* 2011;20(10):1727-36. doi: 10.1007/s11136-011-9903-x [published Online First: 2011/04/12]

28. Rawson KA, Gunstad J, Hughes J, et al. The METER: a brief, self-administered measure of health literacy. *Journal of general internal medicine* 2010;25(1):67-71. doi: 10.1007/s11606-009-1158-7 [published Online First: 2009/11/04]

For peer review only

29. Paiva D, Silva S, Severo M, et al. Cross-cultural adaptation and validation of the health literacy assessment tool METER in the Portuguese adult population. *Patient education and counseling* 2014;97(2):269-75. doi: 10.1016/j.pec.2014.07.024 [published Online First: 2014/08/12]

30. Nasreddine ZS, Phillips NA, Bedirian V, et al. The Montreal Cognitive Assessment, MoCA: a brief screening tool for mild cognitive impairment. *Journal of the American Geriatrics Society* 2005;53(4):695-9. doi: 10.1111/j.1532-5415.2005.53221.x [published Online First: 2005/04/09]

31. Freitas S, Simoes MR, Alves L, et al. Montreal Cognitive Assessment (MoCA): normative study for the Portuguese population. *Journal of clinical and experimental neuropsychology* 2011;33(9):989-96. doi: 10.1080/13803395.2011.589374 [published Online First: 2011/11/16]

32. Pinto P, Severo M, Ruano L, et al. The Cube Test: a novel cognitive screening tool. *Gaceta Sanitaria* 2018;Suppl. 32:108.

33. Ruano L, Sousa A, Severo M, et al. Development of a self-administered web-based test for longitudinal cognitive assessment. *Scientific reports* 2016;6:19114. doi: 10.1038/srep19114 [published Online First: 2016/01/09]

34. Freitas S, Simões MR, Martins C, et al. Estudos de adaptação do Montreal Cognitive Assessment (MoCA) para a população Portuguesa. *Avaliação Psicológica* 2010;9:345-57.

35. Ruano L, Severo M, Sousa A, et al. Tracking cognitive performance in the general population and in patients with mild cognitive impairment with a self-applied computerized test (Brain on Track). *Journal of Alzheimer's Disease* 2019;71(2):541-48.

36. Winblad B, Palmer K, Kivipelto M, et al. Mild cognitive impairment--beyond controversies, towards a consensus: report of the International Working Group on Mild Cognitive Impairment. *Journal of internal medicine* 2004;256(3):240-6. doi: 10.1111/j.1365-2796.2004.01380.x [published Online First: 2004/08/25]

37. American Psychiatric Association. Major neurocognitive disorder. Diagnostic and Statistical Manual of Mental Disorders, Fifth Edition (DSM-5). Arlington, VA: American Psychiatric Association 2013:602-603.

38. Morris JC. The Clinical Dementia Rating (CDR): current version and scoring rules. *Neurology* 1993;43(11):2412-4. [published Online First: 1993/11/01]

39. Kalbfleisch J, Prentice R. The statistical analysis of failure time data. 2nd ed. New York, NY: John Wiley & Sons 2002:203-215.

For peer review only

40. Ruano L, Araújo N, Branco M, et al. Prevalence and Causes of Cognitive Impairment and Dementia in a Population-Based Cohort From Northern Portugal. *American Journal of Alzheimer's Disease & Other Dementiasr* 2018;34(1):49-56. doi: 10.1177/1533317518813550

41. Pereira S, Fontes F, Sonin T, et al. Neurological complications of breast cancer: study protocol of a prospective cohort study. *BMJ open* 2014;4(10):e006301. doi: 10.1136/bmjopen-2014-006301 [published Online First: 2014/10/30]

42. RORENO. Sobrevivência global, Doentes diagnosticados em 2009-10 Região Norte. Porto, Portugal 2017.

43. Monteiro I, Morais S, Costa AR, et al. Changes in employment status up to 5 years after breast cancer diagnosis: A prospective cohort study. *Breast* 2019;48:38-44. doi: 10.1016/j.breast.2019.07.007 [published Online First: 2019/09/08]

44. Gino S, Mendes T, Maroco J, et al. Memory complaints are frequent but qualitatively different in young and elderly healthy people. *Gerontology* 2010;56(3):272-7. doi: 10.1159/000240048 [published Online First: 2009/09/25]

45. Schmand B, Jonker C, Hooijer C, et al. Subjective memory complaints may announce dementia. *Neurology* 1996;46(1):121-5. [published Online First: 1996/01/01]

46. Borkowski JG, Benton AL, Spreen O. Word fluency and brain damage. *Neuropsychologia* 1967;5(2):135-40. doi: 10.1016/0028-3932(67)90015-2

47. Cavaco S, Goncalves A, Pinto C, et al. Semantic fluency and phonemic fluency: regression-based norms for the Portuguese population. *Archives of clinical neuropsychology : the official journal of the National Academy of Neuropsychologists* 2013;28(3):262-71. doi: 10.1093/arclin/act001 [published Online First: 2013/01/24]

48. Babins L, Slater ME, Whitehead V, et al. Can an 18-point clock-drawing scoring system predict dementia in elderly individuals with mild cognitive impairment? *Journal of clinical and experimental neuropsychology* 2008;30(2):173-86. doi: 10.1080/13803390701336411 [published Online First: 2008/10/22]

49. Santana I, Duro D, Freitas S, et al. The Clock Drawing Test: Portuguese norms, by age and education, for three different scoring systems. *Archives of clinical neuropsychology : the official journal of the*

For peer review only

National Academy of Neuropsychologists 2013;28(4):375-87. doi: 10.1093/arclin/act016 [published Online First: 2013/03/23]

50. Duro D, Freitas S, Alves L, et al. O Teste do Desenho do Relógio: influência das variáveis sociodemográficas e de saúde na população portuguesa. *Sinapse* 2012;12(1):5-12.

51. Cavaco S, Goncalves A, Pinto C, et al. Trail Making Test: regression-based norms for the Portuguese population. *Archives of clinical neuropsychology : the official journal of the National Academy of Neuropsychologists* 2013;28(2):189-98. doi: 10.1093/arclin/acs115 [published Online First: 2013/01/10]

52. Army Individual Test Battery. Manual of directions and scoring. Washington, DC: War Department, Adjutant Generals Office 1994.

53. Wechsler D. WMS-III Wechsler Memory Scale-Third Edition. San Antonio, TX: The Psychological Corporation 1997.

54. Weschler D. Escala de Memória de Weschler – 3ª Edição (WMS-III): Manual técnico. Lisboa, Portugal: CEGOC 2008.

55. Wechsler D. WAIS-III Wechsler Adult Intelligence Scale-Third Edition. San Antonio, TX: The Psychological Corporation 1997.

56. Weschler D. Escala de Inteligência de Weschler para Adultos – 3ª Edição (WAIS-III): Manual técnico. Lisboa, Portugal: CEGOC 2008.

57. Fernandes S. Stroop: Teste de Cores e Palavras. Lisboa, Portugal: CEGOC 2013.

58. Golden C. Stroop Colour and Word Test: a manual for clinical and experimental uses. Chicago, IL: Soelting 1978.

59. Guerreiro M. Contributo da neuropsicologia para o estudo das demências. Tese de doutoramento não publicada. Lisboa, Portugal: Faculdade de Medicina de Lisboa 1998.

60. Benedict RH, DeLuca J, Phillips G, et al. Validity of the Symbol Digit Modalities Test as a cognition performance outcome measure for multiple sclerosis. *Multiple sclerosis (Houndmills, Basingstoke, England)* 2017;23(5):721-33. doi: 10.1177/1352458517690821 [published Online First: 2017/02/17]

61. Alves L, Simoes MR, Martins C. The estimation of premorbid intelligence levels among Portuguese speakers: the Irregular Word Reading Test (TeLPI). *Archives of clinical neuropsychology : the official journal of the National Academy of Neuropsychologists* 2012;27(1):58-68. doi: 10.1093/arclin/acr103 [published Online First: 2011/12/06]

For peer review only

62. Campos RC, Gonçalves B. The Portuguese version of the Beck Depression Inventory-II (BDI-II): Preliminary psychometric data with two nonclinical samples. *European Journal of Psychological Assessment* 2011;27(4):258-64. doi: 10.1027/1015-5759/a000072

63. Beck AT, Steer RA, Brown GK. BDI-II Manual. San Antonio, TX: The Psychological Corporation 1996.

64. Mahoney FI, Barthel DW. Funcional evaluation: the Barthel Index. *Maryland state medical journal* 1965;14:61-5. [published Online First: 1965/02/01]

65. Sequeira C. Cuidar de idosos dependentes. Coimbra, Portugal: Quarteto Editora 2007.

66. Lawton MP, Brody EM. Assessment of older people: self-maintaining and instrumental activities of daily living. *The Gerontologist* 1969;9(3):179-86. [published Online First: 1969/01/01]

67. Madureira S, Moleiro C, Verdelho A, et al. Escala de Atividades Instrumentais de Vida Diária (AIVD). In: Simões MR, Santana I, Grupo de Estudos de Envelhecimento Cerebral e Demência, eds. Escalas e Testes na Demência, 3ª Edição. Lisboa, Portugal: Novartis 2015:140-45.

For peer review only

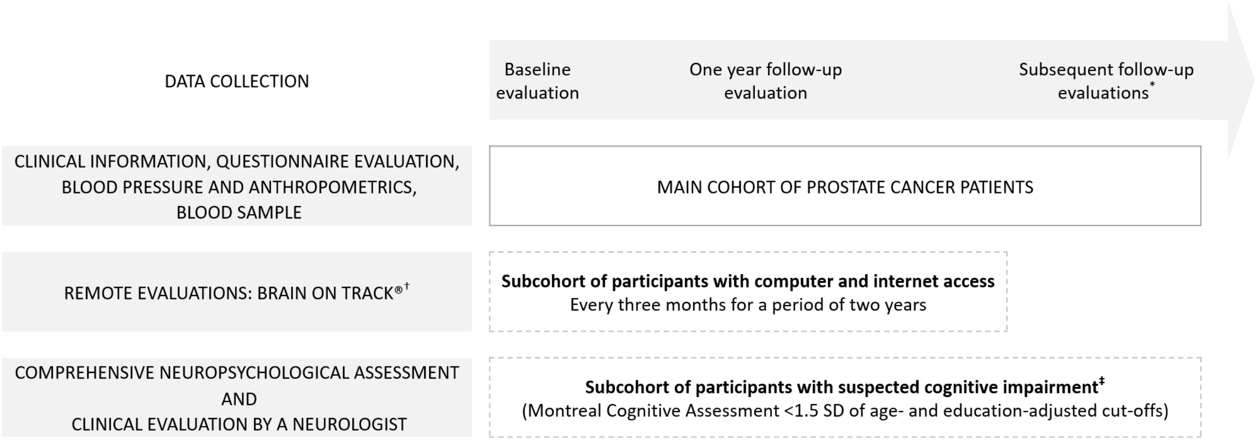

Figure 1. Study design, and timing of baseline and follow-up evaluations in the main cohort and the subcohort of participants with suspected cognitive impairment.

SD – standard deviations.

[*] Subsequent follow-up evaluations will be at three, five, seven and 10 years after the baseline evaluation.

[†] The Brain on Track evaluation will be conducted every three months for a period of two years.

[‡]Only participants who score below 1.5 standard deviations of age- and education-adjusted cut-offs on the Montreal Cognitive Assessment during each evaluation (baseline, and one, three, five, seven and 10 years of follow-up) will be invited for a neuropsychological evaluation where a battery of cognitive tests will be applied. The type of cognitive impairment will be classified through a clinical evaluation performed by a neurologist.

For peer review only

Table 1. Description of the instruments used for the evaluation of all patients.

Instrument	Description	Domains/subscales	Score
**MoCA**[30 34]	Test for the rapid screening of mild cognitive impairment – an intermediate clinical state between normal cognitive aging and dementia.	Attention and concentration; executive functions; memory; language; visuoconstructional skills; calculations; orientation.	Range: 0 to 30<br><br>Higher scores represent better cognitive performance.
**Cube Test**[32]	A two-task cognitive screening tool that consists in completing a 3D cube from six pieces (task 1) and remembering the position of the six pieces on a grid with 25 squares measuring eight by eight centimeters from a previously shown scheme (task 2).	Visuoconstructional skills; executive function; processing speed; delayed memory.	Time to construct the first vertex and to complete cube and the number of pieces correctly assembled in up to six minutes (task 1); number of pieces correctly positioned on the grid (task 2).
**Brain on Track**[33]	A self-administered computerized test intended for longitudinal cognitive testing that includes eight subtests.	Attention; memory; executive functions; language; calculation; constructive ability; visuospatial processing.	Range: virtually unlimited (maximum number of correct answers in a fixed time)<br><br>Higher scores represent better cognitive performance.<br><br>Scores falling below an expected performance threshold for each age/education group; a pattern of decline in individual performance.
**HADS**[20 21]	Scale with 14 questions assessing anxiety and emotional distress among patients during the previous week.	Depression; anxiety.	Range (for each subscale): 0 to 21<br><br>Scores greater than or equal to 11 represent a case of anxiety or depression, as applicable.
**PSQI**[22 23]	Index with 18 questions assessing sleep quality and disturbances during the previous month.	Subjective sleep quality; sleep latency; duration of sleep; habitual sleep efficiency; sleep disorders; use of medications for sleep; daytime dysfunction.	Range: 0 to 21<br><br>Scores greater than five indicate poor sleep quality.
**QLQ-C30**[24 25]	Scale with 30 questions assessing quality of life in patients with cancer during the previous week.	Global health status.<br><br>Functional scales: physical functioning; role functioning; emotional functioning; cognitive functioning; social functioning.<br><br>Symptom scales/items: fatigue; nausea and vomiting; pain; dyspnoea; insomnia; appetite loss; constipation; diarrhoea; financial difficulties.	Range (scales and single-item): 0 to 100<br><br>Higher scores for the global health status and for a functional scale represent a healthy level of quality of life and functioning, respectively.<br><br>Higher scores for a symptom scale/item represents a higher level of symptomatology/ problems.
**QLQ-PR25**[26]	Specific prostate cancer scale with 25 questions assessing quality of life in patients	Functional scales: sexual activity; sexual functioning.	Range (scales and single-item): 0 to 100

For peer review only

	with prostate cancer during the previous week and the last four weeks.	Symptom scales: urinary symptoms; bowel symptoms; hormonal treatment-related symptoms; incontinence aid.	Higher scores for a function scale/item reflect a healthy level of functioning.<br><br>Higher scores for a symptom scale/item reflect a higher level of symptomatology/problems.
**EQ-5D-5L**[27]	A measure of health-related quality of life with five questions and a visual analogue scale.	Mobility, self-care, usual activities, pain/discomfort; anxiety/depression; and a visual analogue scale.	A total of 3125 possible health states are defined to describe the patient's health state. Each state is referred to in terms of a 5-digit code.<br><br>Vertical visual analogue scale<br>Range: 0 to 100<br><br>Higher scores reflect 'The best health you can imagine' and lower scores reflect 'The worst health you can imagine'.
**METER**[28 29]	A measure of health literary including 40 words and 30 non-words.	40 words and 30 non-words.	Range: 0 to 40 and 0 to 30<br><br>Adequate health literacy is defined as scoring at least 35/40 in words and 18/30 in non-words.

MoCA – Montreal Cognitive Assessment; BoT – Brain on Track; HADS – Hospital Anxiety and Depression Scale; PSQI – Pittsburgh Sleep Quality Index; QLQ-C30 – Quality of Life Questionnaire of the European Organization for Research and Treatment of Cancer; QLQ-PR25 – Prostate cancer-specific module of the Quality of Life Questionnaire of the European Organization for Research and Treatment of Cancer; EQ-5D-5L – Measure of health-related quality of life of the EuroQol Group; METER, Medical Term Recognition Test.

For peer review only

Table 2. Description of the battery of instruments used for the neuropsychological evaluation of the subcohort of patients with suspected cognitive impairment*.

Instrument	Description	Cognitive domains/function	Score
**SMC**[44 45]	A 10-item scale regarding subjective memory complaints.	Subjective memory.	Range: 0 to 21<br><br>Higher scores reflect maximal memory complaints.
**Phonemic Verbal Fluency**[46 47]	A test consisting of three trials of one minute each where participants are asked to produce orally as many words as possible beginning with a specific letter.	Executive function; language; semantic memory.	The total trial score corresponds to the number of words correctly produced within one minute. The total test score corresponds to the sum of the three trials.<br><br>Higher scores correspond to better performance.
**18-point CDT**[48-50]	An 18-point clock-drawing scoring system where participants are asked to draw a big circle and put the numbers of the clock, and then they were asked to indicate the time as "10 past 11".	Visuospatial; executive function.	Range: 0 to 18<br><br>Scoring system with three main components: (a) assessment of circle integrity (two points); (b) number placement and sequencing (six points); and (c) placement and size of the hands (six points). Additionally, there are two points for representation of the clock's center and two points for general gestalt.
**TMT**[51 52]	Part A: participants are asked to draw lines to connect 25 randomly positioned numbered circles in numeric order as quickly as possible.<br><br>Part B: participants are asked to draw lines to connect circles in numeric and alphabetic order as quickly as possible, alternating between numbers and letters (progressively up to number 13).	Part A: attention; visual scanning; and speed of eye-hand coordination and information processing.<br><br>Part B: working memory and executive functions; particularly, the ability to switch between sets of stimuli.	Direct measures of performance: time (seconds) to complete part A and part B and performance errors during part A and part B.<br><br>Derived scores: difference score (B - A), ratio score (B/A), proportion score (B - A/A), sum score (A + B), and multiplication score (A × B/100).<br><br>Lower raw scores and higher adjusted scores correspond to better performance.
**WMS-III**[53 54]	Evaluates memory and attention functions using both auditory and visual stimuli.<br><br>A test composed of 17 subtests designed to measure different memory functions in a person with the aim of detecting and discriminating between subcortical vascular dementia and Alzheimer´s disease.	Verbal and visual memories; working memory.	Range:<br>- Immediate recall: 0-50<br>- Delayed recall: 0-50<br>- Auditory recognition: 0-30<br>- Visual reproduction: 0-104<br>- Digit span: 0-30

For peer review only

	Subtests used: Logical Memory I, II; Visual Reproduction I, II; Digit Span		Higher scores correspond to better performance.
**WAIS-III**[55 56]	Measures intelligence and cognitive ability in adults and older adolescents.<br><br>Subtests used: Digit-Symbol-Coding which consists of digit-symbol pairs followed by a list of digits and under each digit the participants write down the corresponding symbol as fast as possible; and Symbol Search, in which, the participants are asked to look at two groups of symbols and to indicate if any of the symbols of the first group are present in the other group.	Attention/concentration; executive function (sequencing); motor function; processing speed.	The number of correct symbols within the allowed time (120 seconds) is measured.
**Stroop Test**[57 58]	Assesses the ability to inhibit cognitive interference, which occurs when the processing of a stimulus feature affects the simultaneous processing of another attribute of the same stimulus.<br><br>This test has three trials: 1) the participant is required to read the colour names printed in black ink as quickly as possible; 2) the participant is required to name the colour of colored dots as quickly as possible; 3) the participant is required to name the colour of the ink of the colour name words (the colour name does not match the colour of the ink).	Executive functions (inhibitory control); selective attention.	Scores for each trial indicate the number of correct responses. An interference score can be generated that quantifies the participant's ability to inhibit the inappropriate response of reading the colour name as opposed to the colour of the ink used to print the colour name in the third trial.
**Token Test – short form**[59]	A test designed to assess the comprehension of commands that vary in degree of linguistic difficulty but which are relatively independent of defects in other aspects of intellectual capacity such as memory and vocabulary.<br><br>The test consists of six subsections which represent different levels of linguistic difficulty. The participant is presented with tokens of different shapes (i.e., circles, squares, triangles), sizes, and colours, and is required to perform certain acts with the tokens, such as point to selected tokens,	Attention and vigilance; verbal functions.	Range: 0-36

For peer review only

	touch them, pick them up, and place one token on top of another.		
**SDMT**[60]	A quick screening test for organic cerebral dysfunction.<br><br>The test involves a simple substitution task that can be easily performed: using a reference key, the participant has 90 seconds to pair specific numbers with given geometric figures.	Organic cerebral dysfunction.	Individuals with cerebral dysfunction perform poorly.
**TeLPI**[61]	A Portuguese irregular word reading test using 46 irregular, infrequent Portuguese words designed to assess premorbid intelligence.	Premorbid Intelligence Quotient (IQ): full scale IQ; Verbal IQ; performance IQ	Range: number of errors (maximum of 46) and years of education are inserted in three linear equations to calculate the three types of IQ
**BDI-II**[62 63]	A 21 question measure assessing the presence of depressive symptoms experienced by the participant within the past week.	Emotional functioning.	Range: 0 to 63<br><br>A cut-off score indicative of mild depressive symptoms is greater than 10 and for severe depressive symptoms is greater than 30.
**Barthel ADL Index**[64 65]	An index to measure functional disability, focused on bodily oriented personal care.	Functional domains: feeding; incontinence; transferring; toileting; dressing; bathing.	Range: 0 to 100<br><br>Lower scores reflect increased disability.
**IADL**[66 67]	An eight item scale used to assess independent living skills which include more complex activities (i.e. "instrumental activities of daily living") necessary for functioning in community settings.	Functional domains: using the telephone; shopping; food preparation; housekeeping; laundry; transport; medication; finances.	Range: 0 to 8<br><br>Higher scores reflect high function, independence.

SMC – Subjective Memory Complains scale; CDT – Clock Drawing Test; TMT – Trail Making Test; WMS-III – Wechsler Memory Scale – Third Edition; WAIS-III – Wechsler Adult Intelligence Scale – Third Edition; SDMT – Symbol and Digit Modalities Test; TeLPI – Irregular Word Reading Test; BDI-II – Beck Depression Inventory – Second Edition; Barthel ADL Index – Barthel Activities of Daily Living Index; IADL – Lawton and Brody Instrumental Activities of Daily Living.

* Only patients who score below 1.5 standard deviations of age- and education-adjusted cut-offs at the Montreal Cognitive Assessment during each evaluation (baseline, and one, three, five, seven and 10 years of follow-up) will be invited for a neuropsychological evaluation.

For peer review only

BMJ Open

Cognitive decline in prostate cancer patients: study protocol of a prospective cohort – NEON-PC

Journal:	*BMJ Open*
Manuscript ID	bmjopen-2020-043844.R1
Article Type:	Protocol
Date Submitted by the Author:	23-Nov-2020
Complete List of Authors:	Araujo, Natalia; UP EPIUnit, Instituto de Saúde Pública da Universidade do Porto<br>Morais, Samantha; UP EPIUnit, Instituto de Saúde Pública da Universidade do Porto; FMUP, Faculdade de Medicina da Universidade do Porto, Departamento de Ciências da Saúde Pública e Forenses e Educação Médica<br>Costa, Ana ; UP EPIUnit, Instituto de Saúde Pública da Universidade do Porto<br>Braga, Raquel; UP EPIUnit, Instituto de Saúde Pública da Universidade do Porto; Unidade Local de Saúde de Matosinhos EPE<br>Carneiro, Ana; IPO-PORTO, Instituto Português de Oncologia do Porto Francisco Gentil, EPE, Serviço de Oncologia<br>Cruz, Vitor; UP EPIUnit, Instituto de Saúde Pública da Universidade do Porto; Unidade Local de Saúde de Matosinhos EPE, Serviço de Neurologia<br>Ruano, Luis; UP EPIUnit, Instituto de Saúde Pública da Universidade do Porto; Centro Hospitalar de Entre o Douro e Vouga EPE, Serviço de Neurologia<br>Oliveira, Jorge; IPO-PORTO, Instituto Português de Oncologia do Porto Francisco Gentil, EPE, Serviço de Urologia<br>Figueiredo, Luis; Universidade do Minho Instituto de Investigação em Ciências da Vida e Saúde, Escola de Medicina da Universidade do Minho; Centro Hospitalar de São João EPE, Serviço de Urologia<br>Pereira, Susana; UP EPIUnit, Instituto de Saúde Pública da Universidade do Porto; IPO-PORTO, Instituto Português de Oncologia do Porto Francisco Gentil, EPE, Serviço de Neurologia<br>Lunet, Nuno; FMUP, Faculdade de Medicina da Universidade do Porto, Departamento de Ciências da Saúde Pública e Forenses e Educação Médica; UP EPIUnit, Instituto de Saúde Pública da Universidade do Porto
**Primary Subject Heading**:	Epidemiology
Secondary Subject Heading:	Oncology, Neurology, Urology
Keywords:	Neurological oncology < NEUROLOGY, Epidemiology < ONCOLOGY, Urological tumours < ONCOLOGY, Delirium & cognitive disorders < PSYCHIATRY

For peer review only - http://bmjopen.bmj.com/site/about/guidelines.xhtml

I, the Submitting Author has the right to grant and does grant on behalf of all authors of the Work (as defined in the below author licence), an exclusive licence and/or a non-exclusive licence for contributions from authors who are: i) UK Crown employees; ii) where BMJ has agreed a CC-BY licence shall apply, and/or iii) in accordance with the terms applicable for US Federal Government officers or employees acting as part of their official duties; on a worldwide, perpetual, irrevocable, royalty-free basis to BMJ Publishing Group Ltd ("BMJ") its licensees and where the relevant Journal is co-owned by BMJ to the co-owners of the Journal, to publish the Work in this journal and any other BMJ products and to exploit all rights, as set out in our licence.

The Submitting Author accepts and understands that any supply made under these terms is made by BMJ to the Submitting Author unless you are acting as an employee on behalf of your employer or a postgraduate student of an affiliated institution which is paying any applicable article publishing charge ("APC") for Open Access articles. Where the Submitting Author wishes to make the Work available on an Open Access basis (and intends to pay the relevant APC), the terms of reuse of such Open Access shall be governed by a Creative Commons licence – details of these licences and which Creative Commons licence will apply to this Work are set out in our licence referred to above.

Other than as permitted in any relevant BMJ Author's Self Archiving Policies, I confirm this Work has not been accepted for publication elsewhere, is not being considered for publication elsewhere and does not duplicate material already published. I confirm all authors consent to publication of this Work and authorise the granting of this licence.

For peer review only

Cognitive decline in prostate cancer patients: study protocol of a prospective cohort – NEON-PC

Natália Araújo[1], Samantha Morais[1,2], Ana Rute Costa[1], Raquel Braga[1,3], Filipa Carneiro[4], Vítor Tedim Cruz[1,5], Luís Ruano[1,6], Jorge Oliveira[4], Luís Pacheco-Figueiredo[7,8], Susana Pereira[1,4], Nuno Lunet[1,2]

[1] EPIUnit – Instituto de Saúde Pública, Universidade do Porto, Porto, Portugal

[2] Departamento de Ciências da Saúde Pública e Forenses e Educação Médica, Faculdade de Medicina da Universidade do Porto, Porto, Portugal

[3] Unidade Local de Saúde de Matosinhos, Matosinhos, Portugal

[4] Instituto Português de Oncologia do Porto, Porto, Portugal

[5] Serviço de Neurologia, Unidade de Saúde Local de Matosinhos, Matosinhos, Portugal

[6] Serviço de Neurologia, Centro Hospitalar Entre Douro e Vouga, Santa Maria da Feira, Portugal

[7] Instituto de Investigação em Ciências da Vida e Saúde, Escola de Medicina da Universidade do Minho, Braga, Portugal

[8] Serviço de Urologia, Centro Hospitalar São João, Porto, Portugal

Corresponding author:

Nuno Lunet – nlunet@med.up.pt

Departamento de Ciências da Saúde Pública e Forenses e Educação Médica, Faculdade de Medicina da Universidade do Porto

Al. Prof. Hernâni Monteiro

4200-319 Porto, Portugal

Phone: +351 225 513 652; Fax: +351 225 513 653

For peer review only

Abstract

Introduction: Prostate cancer is the most prevalent oncologic disease among men in industrialized countries. Despite the high survival rates, treatments are often associated with adverse effects, including metabolic and cardiovascular complications, sexual dysfunction and, to a lesser extent, cognitive decline. This study was primarily designed to evaluate the trajectories of cognitive performance in prostate cancer patients, and to quantify the impact of the disease and its treatments on the occurrence of cognitive decline.

Methods: Participants will be recruited from two main hospitals providing care to approximately half the prostate cancer patients in Northern Portugal (Portuguese Institute of Oncology of Porto and São João Hospital Centre), and will comprise a cohort of recently diagnosed prostate cancer patients proposed for different treatment plans, including: 1) radical prostatectomy; 2) brachytherapy and/or radiotherapy; 3) radiotherapy in combination with androgen deprivation therapy; 4) androgen deprivation therapy (with or without chemotherapy). Recruitment began in February 2018 and is expected to continue until the first semester of 2021. Follow-up evaluations will be conducted at one, three, five, seven and 10 years. Sociodemographic, behavioral and clinical characteristics, anxiety and depression, health literacy, health status, quality of life, and sleep quality will be assessed. Blood pressure and anthropometrics will be measured, and a fasting blood sample will be collected. Participants' cognitive performance will be evaluated before treatments and throughout follow-up (Montreal Cognitive Assessment and Cube Test as well as Brain on Track for remote monitoring). All participants suspected of cognitive impairment will undergo neuropsychological tests and clinical observation by a neurologist.

Ethics and dissemination: The study was approved by the Ethics Committee of the hospitals involved. All participants will provide written informed consent, and study procedures will be developed to ensure data protection and confidentiality. Results will be disseminated through publication in peer-reviewed journals and presentation in scientific meetings.

For peer review only

Article Summary

Strengths and limitations of this study

- This protocol describes a prospective cohort study of prostate cancer patients, expected to reflect the contemporary patterns of diagnosis and treatment in developed countries.
- Cognitive impairment will be characterized regarding its severity and possible aetiologies through neuropsychological and clinical evaluations.
- Short- and long-term effects as well as mediators of the effect of androgen deprivation therapy on cognitive performance will be analysed.
- A longitudinal remote monitoring tool of cognitive function will be used, in addition to state-of-the-art methods, which allows for more frequent standardized evaluations, while reducing learning effects of repeated measurements.
- Only a measure of overall cognitive function will be obtained from all participants and multiple cognitive domains will only be evaluated in patients with probable cognitive impairment.

For peer review only

For peer review only - http://bmjopen.bmj.com/site/about/guidelines.xhtml

Introduction

Prostate cancer is the second most common neoplasm and the fifth leading cause of death from cancer among men, with an estimated 1.3 million new cases and 359 thousand deaths in 2018 worldwide.[1] In recent decades, prostate cancer incidence has been heavily influenced by diagnoses following prostate-specific antigen testing of asymptomatic individuals and by the detection of latent cancer in tissue removed during prostatectomy or autopsy.[1] At the same time, prostate cancer mortality has been decreasing in many countries, which has been linked to earlier diagnosis because of extensive use of prostate-specific antigen screening, and improved treatment including radical prostatectomy, hormonal therapy and radiation therapy.[2 3] Increases in prostate cancer survival[4] require a comprehensive assessment of the burden of cancer, due to the disease, treatment and sequelae.[5 6]

Androgen deprivation therapy is used in the treatment of approximately half of all prostate cancer patients,[7 8] and it may last from six to 36 months, on an intermittent basis or continue indefinitely.[9] The use of androgen deprivation therapy and its impact on cognitive function has been assessed, both in prospective studies evaluating cognitive performance using neuropsychological tests and in large retrospective studies reporting the risk of dementia or of Alzheimer's disease in prostate cancer patients according to androgen deprivation therapy exposure.[10-12] However, methodological heterogeneity does not allow for the direct comparison of results, and shortcomings of the study designs, including small sample sizes, short follow-up periods or limited quality of information on cognitive status, as well as residual confounding, preclude more robust conclusions on this topic.[10] Also, in addition to the possible direct effect of androgen deprivation therapy on cognitive function due to the drop in serum testosterone and its biological activity in certain areas of the brain,[13] hormonal changes may also cause metabolic alterations,[14] with an increase in cardiovascular risk factors, such as an increase in insulin resistance, serum cholesterol and triglycerides, or anemia,[15] which in turn are associated to cognitive decline.[16-19] This possible indirect effect may take longer to manifest at the brain than the direct decrease in testosterone serum levels, and it may be related to the development of dementia. The potential mediator effect of these biochemical and hematologic parameters has not been studied. Prospective investigations including an accurate characterization of the cognitive performance of prostate cancer patients proposed

For peer review only - http://bmjopen.bmj.com/site/about/guidelines.xhtml

for different types of treatment, and analyses accounting for distinct causal pathways may contribute to a better understanding of the effects of prostate cancer and its treatments on cognitive decline.

Therefore, this project primarily aims to understand the impact of androgen deprivation therapy on the cognitive performance of prostate cancer patients in Northern Portugal. The main specific objectives are as follows:

1) To describe the trajectories of cognitive performance over time (up to 10 years) in prostate cancer patients under different treatments and, in comparison to the general population, by using the Montreal Cognitive Assessment tool, the Cube Test and Brain on Track®. The relation between patients' characteristics, cancer treatments and different cognitive trajectories will also be assessed.

2) To quantify the association between androgen deprivation therapy and cognitive decline, in the short- and in the long-term.

3) To assess the role of metabolic syndrome and anemia as possible mediators of the androgen deprivation therapy effect on cognitive performance.

Methods and analysis

We describe a prospective cohort study that will evaluate prostate cancer patients selected among those being treated at the two largest hospitals providing cancer care in the North of Portugal, which attend half the prostate cancer patients in this region. Recruitment started in February 2018 and is ongoing. We expect to complete it in the first semester of 2021.

Eligibility criteria

Eligible participants are those with a recent diagnosis of prostate cancer and being initially proposed for radical prostatectomy (group 1), brachytherapy or radiotherapy (group 2), radiotherapy in combination with androgen deprivation therapy (group 3), or androgen deprivation therapy with or without chemotherapy (group 4), and prostate cancer survivors never treated with androgen deprivation therapy

For peer review only

before, who present with a recurrence of the disease to be treated with androgen deprivation therapy, with or without chemotherapy (group 5).

Participants who had a previous chemotherapy or radiotherapy treatment for another primary cancer, or a diagnosis of a psychiatric or a neurologic condition impairing cognitive function before the prostate cancer diagnosis, or being unable to understand the purpose of the study or to collaborate will be excluded, as well as those expected to receive cancer treatments outside the Portuguese Institute of Oncology of Porto or the São João Hospital Centre.

Participants' recruitment and follow-up

Prostate cancer patients will be consecutively recruited at the Portuguese Institute of Oncology of Porto and the São João Hospital Centre, from February 2018 to the first semester of 2021. Participants will be evaluated at baseline (before any treatment for recently diagnosed patients or before androgen deprivation therapy for patients with a recurrence of the disease), and at one, three, five, seven and 10 years after enrolment, as depicted in Figure 1.

Data collected from medical records

Clinical characteristics, including co-morbidities, medication and cancer treatment (including all drugs used for systemic treatment of prostate cancer, either at initial or follow-up treatments, and duration), as well as prognostic and treatment response biomarkers will be systematically collected by medical doctors from the patients´ medical records. Prostate cancer staging will be in accordance with the American Joint Committee on Cancer TNM system classification[20] and risk stratification according to the National Comprehensive Cancer Network (www.nccn.org).

Questionnaire evaluation

Data on sociodemographic (birth date, address, marital status, education, occupation), lifestyle and dietary characteristics (smoking and alcohol consumption, and intake of fruits and vegetables, physical

For peer review only - http://bmjopen.bmj.com/site/about/guidelines.xhtml

activity and sedentary behaviours) will be collected through questionnaires applied by a trained interviewer. Anxiety and depression,[21 22] sleep quality,[23 24] quality of life and health status,[25-28] and health literacy[29 30] will be evaluated through self-administered questionnaires validated for the Portuguese population, and are described in detail in Table 1.

Blood pressure

Blood pressure will be measured with a digital blood pressure monitor (Omron M6). Participants will be asked to remain seated, with the right arm and back supported and feet firmly on the floor, and to abstain from speaking during the entire procedure. The cuff will be placed on the right arm so the bottom margin is approximately two to three centimeters above the antecubital fossa. A larger or a smaller cuff will be used as necessary to fit the arm of the participant. Three measurements with one-minute intervals will be registered.

Anthropometrics

Weight and height will be measured with participants in light clothes and no shoes, and registered to the nearest kilogram and centimeter, respectively, using a digital column scale (Seca 799). Waist and hip perimeters will be measured using a non-elastic measuring tape (Seca 211) with participants standing, with feet slightly apart and the arms relaxed along the body; waist perimeter will be measured at half the distance between the last rib and the iliac crest. Hip perimeter will be measured with participants in the same position, with the measuring tape placed at the widest part of the hip below the iliac crest. Both waist and hip circumferences will be registered to the nearest millimeter. Most measurements are expected to be performed in the morning.

Blood sample

A fasting blood sample (at least 12 hours) will be collected by the hospitals´ nurses using venous puncture, and blood samples will be centrifuged at 3000 rpm for 10 minutes to obtain plasma and serum, within 30 to 60 minutes. Total cholesterol, high density lipoprotein cholesterol, triglycerides, glycaemia, glycated

For peer review only - http://bmjopen.bmj.com/site/about/guidelines.xhtml

hemoglobin and hemoglobin will be analysed. Plasma and serum samples will be stored in small aliquots at -80º C until the end of the study (10 years).

For peer review only

For peer review only - http://bmjopen.bmj.com/site/about/guidelines.xhtml

Cognitive function evaluation

Cognitive function will be evaluated using the Montreal Cognitive Assessment[31 32] and the Cube Test,[33] at baseline and at each of the subsequent follow-up evaluations, and with a web-based tool for remote longitudinal assessment (Brain on Track),[34] every three months for a period of up to 10 years.

Participants suspected of cognitive impairment will undergo a comprehensive neuropsychological assessment that will allow specific domains of cognitive function to be analysed; the battery of tests is described in Table 2. Additionally, those with confirmed cognitive impairment will undergo a clinical evaluation by a neurologist.

<u>The Montreal Cognitive Assessment</u>

The Montreal Cognitive Assessment is a pen and paper screening test, developed to detect mild cognitive impairment. It assesses eight cognitive domains (visuospatial ability, executive function, attention, concentration, working memory, language, verbal memory and orientation), generating a total score ranging from 0 to 30.[31] The translated, culturally adapted and validated version of the Montreal Cognitive Assessment for the Portuguese population[35] will be used, and the performance of participants will be classified as probable cognitive impairment when the score is 1.5 standard deviations below the mean of age- and education-based group distribution from published normative data.[32]

<u>The Cube Test</u>

The Cube Test will be used as a rapid cognitive screening tool, which can be applied to illiterate participants, or those with low educational levels, language or hearing deficits.[33] The Cube Test is easy to apply, and the simple instructions and scoring procedures contribute for standardized use. The test is based on the time spent in assembling the six faces of a 3D cube and then, the correct placement of the six faces of the 3D cube on a grid with 25 squares measuring eight by eight centimetres. The Cube Test assesses visuoconstructive, visuospatial and executive functions, visuospatial working memory, information processing speed, incidental learning, motor processing speed, and manual dexterity.

For peer review only - http://bmjopen.bmj.com/site/about/guidelines.xhtml

Brain on Track

The Brain on Track test will be used for the remote evaluation of changes in cognitive function over a two-year period. This is a computerised cognitive monitoring test, which was developed and validated in the Portuguese population, showing good internal consistency, discriminative ability and reliability.[34 36] Brain on Track evaluates different cognitive domains, including attention, memory, executive functions, language, calculation, constructive capacity and visuospatial processing, through 11 exercises designed to include random elements and alternate sequences to lower the learning effect of repeating cognitive tests. It is to be performed using a home computer to access a web platform where different cognitive tests are uploaded. Each patient´s results are stored and can be monitored by the research team.

Patients will be eligible to participate if they have completed at least three years of schooling, have no severe motor, visual or language impairments that prevent cognitive assessment, have easy access to a computer with an internet connection, and are able to use a computer without help. At the end of the baseline evaluation, participants will undergo a training session, and will be instructed to remotely log into the web platform and proceed to the first evaluation after one week, and then every three months for a period of two years. A Short Message Service reminder will be sent to participants one day before each remote evaluation. The research team will be automatically notified when participants fail to perform the test in order to re-schedule the evaluation.

Comprehensive neuropsychological assessment and clinical evaluation by a neurologist

All participants who score below 1.5 standard deviations of age- and education-adjusted cut-offs on the Montreal Cognitive Assessment[31 32] in each evaluation (baseline, and one, three, five, seven and 10 years of follow-up) will undergo a neuropsychological evaluation, expectedly within one-month, comprising a battery of cognitive tests (Table 2). The type and progressive nature of cognitive impairment, and its functional impact will be determined through a clinical evaluation performed by a neurologist, with a close surrogate present. Additionally, participants with a first neuropsychological evaluation will be re-assessed with the same battery of tests, independently of their Montreal Cognitive Assessment score in the subsequent follow-up evaluations. A random sample of 30 patients with normal scores on the Montreal

For peer review only - http://bmjopen.bmj.com/site/about/guidelines.xhtml

Cognitive Assessment will also perform a neuropsychological evaluation at one, three, five, seven and ten years of follow-up, as a control group.

Participants will be classified as having mild cognitive impairment when presenting cognitive complaints over a period of at least six months, as reported by the patient or family members, modest cognitive decline from a previous level of performance reported by the patient or family members, and neuropsychological evaluation scores at least 2.0 standard deviations below the age-corrected norms in at least one cognitive domain or at least 1.5 standard deviations below the age-corrected norms in at least two cognitive domains, while also presenting no clinical depression or interference of cognitive function with independence in daily activities.[37 38]

Dementia will be defined according to the criteria used for defining major neurocognitive disorder of the *Diagnostic and Statistical Manual of Mental Disorders* (fifth edition), that is significant cognitive impairment in at least one cognitive domain representing a significant decline from a previous level of functioning that interferes with independence in daily activities.[38] The severity of dementia will be classified using the clinical dementia rating scale.[39] The initial clinical classification will be confirmed after at least six months of clinical follow-up by a neurologist, and a complete diagnostic workup to identify other potential causes of cognitive impairment not related to oncologic disease, including blood analyses for treatable causes of dementia and imaging studies.

Data analyses and sample size

The frequency of cognitive decline and impairment will be described in the different categories of socio-demographic (age, education, employment, marital status) and lifestyle (alcohol intake, tobacco smoking, physical activity, fruit and vegetables consumption) variables, as well as patient reported outcomes (anxiety, depression, quality of sleep), and according to the clinical characteristics of prostate cancer (cancer stage, risk strata) as well as treatments.

Trajectories of cognitive decline will be described through indicators of cognitive performance at different moments of evaluation using the appropriate format according to the nature of the variables and their distribution. Fixed- and mixed-effects models will be computed to compare cognitive performance

For peer review only - http://bmjopen.bmj.com/site/about/guidelines.xhtml

trajectories (considering age and education) over time, according to other sociodemographic and clinical characteristics, for each of the treatment groups.

Prevalence at baseline and incidence measures (incidence rates and cumulative incidences) and the corresponding 95% confidence intervals will be estimated to quantify the frequency of cognitive impairment, and the association between treatments and incident cognitive impairment. Cumulative incidence will be estimated considering death as a competitive event, according to the Kalbfleisch and Prentice method.[40] Crude and adjusted relative risks will be calculated.

The sample size was calculated considering the objective to quantify the association between the use of androgen deprivation therapy and cognitive decline between the baseline and the one-year evaluation, defined as a variation in the score from baseline to the one-year evaluation below 1.5 standard deviations of the distribution in the cohort, of the changes in cognitive scores over the same time period. For this, assuming a statistical power of 80%, a level of significance of 5% and a 1:1 ratio between androgen deprivation therapy-exposed (groups 3 and 4) and unexposed (groups 1 and 2), 600 prostate cancer patients will be necessary to detect a two-fold higher proportion of participants (14%) with cognitive decline in the androgen deprivation therapy group. Secondary analyses will be conducted considering the exposure to each specific hormonal treatment.

For the description of cognitive performance trajectories, and the calculation of the prevalence of cognitive impairment at baseline and incidence measures (incidence rates and cumulative incidences), the sample size will influence the precision of the estimates at each moment but will not be a limiting factor for the essentially descriptive accomplishment of these objectives. Nevertheless, considering the prevalence of cognitive impairment in the general population of Northern Portugal of 9.6%,[41] a precision of 2.4%, and a 95% confidence level, a sample of 579 individuals will be needed. As such, the estimated sample size calculated above will also be sufficient for estimating the prevalence of cognitive impairment in the population of prostate cancer patients.

Considering the high potential for confounding by indication, propensity scores calculated based on several disease characteristics, including prognostic biomarkers and predictors of response to treatment,

For peer review only - http://bmjopen.bmj.com/site/about/guidelines.xhtml

will be used in data analysis. Causal diagrams will be used to support the decisions regarding the potential role of the different sociodemographic, lifestyle, clinical and treatment variables in the causal pathways.

Training of interviewers and the use of standardized procedures for data collection are expected to contribute to a low proportion of missing data, and no imputation is being planned.

Considering our experience in another cancer cohort,[42] we estimate that approximately a third of the total sample will participate in the Brain on Track evaluation. Using as criteria for referral of participants to the comprehensive neuropsychological assessment, the Montreal Cognitive Assessment cut-off score, 1.5 standard deviations below the mean of age and education-based group distribution from published normative data[32], we expect at least 42 patients to undergo a neuropsychological assessment at baseline and at each subsequent evaluation.

Taking into account the survival of prostate cancer patients in the North of Portugal,[43] and the high participation obtained in a previous prospective cohort study of breast cancer patients;[42 44] we estimate that at least 90% and 80% of patients will participate in the one- and five-year follow-up evaluations, respectively. In order to minimize refusals and losses to follow-up, all evaluations will be scheduled to take place on the same day as routine appointments in the respective hospital and participants will be invited again when they miss scheduled appointments.

Contingency plan

Due to the COVID-19 pandemic, recruitment and evaluation of participants were interrupted from March to June 2020. Beginning in July, procedures were adapted to minimize the risk of infection for participants and members of the research team. Only the Montreal Cognitive Assessment and the neuropsychological evaluation will be performed face-to-face at the hospital. Participants will answer the questionnaire on sociodemographic, and lifestyle and dietary characteristics during a telephone interview. Self-administered questionnaires will be completed at home and sent mailed back with a pre-paid envelope.

Anthropometrics measurements, blood sample collection and the Cube test evaluation will not be performed. Weight, height, blood pressure and blood sample parameters will be retrieved from medical

For peer review only - http://bmjopen.bmj.com/site/about/guidelines.xhtml

records when available or asked to the participants. The initial training session for the Brain on Track evaluation will be conducted through videoconference.

The impact of the pandemic on the course of this investigation, namely regarding participation and retention rates, completeness of information and potential losses of validity and precision will be addressed specifically. Additional mitigation measures may have to be adopted, namely an extension of the recruitment period or an increase in the sample size.

Patient and Public Involvement

Patients and public were not involved in the conception, design, and dissemination of this study.

Ethics and dissemination

Ethics approval was obtained from the Ethics Committees of the Portuguese Institute of Oncology of Porto (Ref. CES 89/017) and the São João Hospital Centre (Ref. 76/17) and by the Portuguese Data Protection Authority (Authorization 3478/2017). Written informed consent will be obtained from all participants after the project´s aims and procedures are fully explained by a member of the research team.

This is an observational study in which prostate cancer patients will be followed according to usual clinical practice, as such the occurrence of harmful effects related to participation in the study are not expected. Participants will receive detailed information about the research purpose and objectives, name and institution of the researchers, expected duration of the interview, voluntary nature of participation, clearly stating that there will be no penalty for those who refuse to participate, and ensuring confidentiality and anonymity of all the information provided. Participants will be asked to give authorization for collection of data from their personal clinical records. After clarification of any doubts, an informed consent will be signed in duplicate and a copy will be given to each participant. All participants will be informed that they can leave the study at any time, and this decision does not affect their medical care. There is no expected risk or discomfort other than those arising from interviewing, collecting venous blood samples and physical measurements (height, weight, blood pressure). Only participants able to

For peer review only - http://bmjopen.bmj.com/site/about/guidelines.xhtml

For peer review only

understand the study and provide informed consent will be included. To minimize possible discomfort due to the required trips to the hospital for face-to-face evaluations or the duration of interviews, and to avoid unnecessary burden and travel expenses, data collection procedures were designed to last no more than 60 minutes, and will be scheduled to take place on the same day as other appointments in the respective hospitals as part of regular clinical care, preferably in the morning due to the fasting requirement. Starting in July 2020, only the Montreal Cognitive Assessment will be performed face-to-face at the hospital, to reduce the risk of infection by the severe acute respiratory syndrome coronavirus 2 (SARS-CoV-2).

This study requires the collection and processing of sensitive personal data including health and clinical data from questionnaires and the clinical files of patients. Therefore, additional measures will be taken to protect the anonymity and the confidentiality of all participants. All data regarding clinical aspects will be collected by clinical members of the research team and privacy is assured. All participants will have a study-specific identification number, which will be used in all questionnaires and stored blood samples. The correspondence between this identification number and the personal identifiable information will be stored in a file, to which only the principal investigator will have access. Only the research team will have access to the database with anonymized data, saved on a password-protected secure computer. No personal identifiers will be used in data analyses. The same procedures will be adopted for each of the evaluations.

The expected results may contribute to elucidate the magnitude of the androgen deprivation therapy effect on the cognitive function of prostate cancer patients, and the possible mediator effect of metabolic syndrome and anemia in this process. This may help clinical decisions regarding the pharmacological class to be used in patients more vulnerable to cognitive impairment. This study may also contribute to the refinement and validation of the longitudinal monitoring tool Brain on Track. Considering the 10-year temporal horizon of this project, the follow-up of the cohort assembled will contribute to a better understanding of the long-term trajectories of cognitive performance and the iatrogenic effects of prostate cancer treatments.

For peer review only

The findings of this project will be submitted for publication in international peer-reviewed journals, and proposed for presentation in relevant national and international conferences, which will allow for the dissemination of the main findings across the medical community. Press releases through mass media will also be issued to promote the dissemination of information relevant to the general population and policy makers. Furthermore, the project will contribute to the training of researchers through the production of masters´ theses and doctoral dissertations.

For peer review only

Authors´ contributions

NL and SP conceived and designed the study. NA wrote the first version of the manuscript. ARC, FC, JO, LPF, LR, NL, RB, SM, SP and VTC critically revised the manuscript for relevant intellectual content. All authors approved the final version for submission.

Funding statement

This study was funded by FEDER through the Operational Programme Competitiveness and Internationalization and national funding from the Foundation for Science and Technology – FCT (Portuguese Ministry of Science, Technology and Higher Education) under the project "NEON-PC - Neuro-oncological complications of prostate cancer: longitudinal study of cognitive decline" (POCI-01-0145-FEDER-032358; Ref. PTDC/SAU-EPI/32358/2017), and the Unidade de Investigação em Epidemiologia - Instituto de Saúde Pública da Universidade do Porto (EPIUnit) (UIDB/04750/2020) financed by national funds from FCT. SM was funded under the scope of the project "NEON-PC - Neuro-oncological complications of prostate cancer: longitudinal study of cognitive decline" (POCI-01-0145-FEDER-032358; Ref. PTDC/SAU-EPI/32358/2017). Individual PhD grants attributed to ARC (SFRH/BD/102181/2014) and NA (SFRH/BD/119390/2016) were funded by FCT and the "Programa Operacional Capital Humano" (POCH/FSE).

Competing interests statement

None declared.

For peer review only - http://bmjopen.bmj.com/site/about/guidelines.xhtml

References

1. Bray F, Ferlay J, Soerjomataram I, et al. Global cancer statistics 2018: GLOBOCAN estimates of incidence and mortality worldwide for 36 cancers in 185 countries. *CA: a cancer journal for clinicians* 2018 doi: 10.3322/caac.21492 [published Online First: 2018/09/13]

2. Fontes F, Severo M, Castro C, et al. Model-based patterns in prostate cancer mortality worldwide. *British journal of cancer* 2013;108(11):2354-66. doi: 10.1038/bjc.2013.217 [published Online First: 2013/05/11]

3. Wong MC, Goggins WB, Wang HH, et al. Global incidence and mortality for prostate cancer: analysis of temporal patterns and trends in 36 countries. *European urology* 2016;70(5):862-74. doi: 10.1016/j.eururo.2016.05.043 [published Online First: 2016/06/13]

4. Trama A, Foschi R, Larranaga N, et al. Survival of male genital cancers (prostate, testis and penis) in Europe 1999-2007: results from the EUROCARE-5 study. *European journal of cancer (Oxford, England : 1990)* 2015;51(15):2206-16. doi: 10.1016/j.ejca.2015.07.027 [published Online First: 2015/10/01]

5. Treanor CJ, Li J, Donnelly M. Cognitive impairment among prostate cancer patients: an overview of reviews. *European journal of cancer care* 2017;26(6) doi: 10.1111/ecc.12642 [published Online First: 2017/02/09]

6. McGinty HL, Phillips KM, Jim HS, et al. Cognitive functioning in men receiving androgen deprivation therapy for prostate cancer: a systematic review and meta-analysis. *Supportive care in cancer : official journal of the Multinational Association of Supportive Care in Cancer* 2014;22(8):2271-80. doi: 10.1007/s00520-014-2285-1 [published Online First: 2014/05/27]

7. Shahinian VB, Kuo YF, Freeman JL, et al. Increasing use of gonadotropin-releasing hormone agonists for the treatment of localized prostate carcinoma. *Cancer* 2005;103(8):1615-24. doi: 10.1002/cncr.20955 [published Online First: 2005/03/03]

8. Barry MJ, Fowler FJ, Jr., O'Leary M P, et al. The American Urological Association Symptom Index for Benign Prostatic Hyperplasia. *The Journal of urology* 2017;197(2S):S189-S97. doi: 10.1016/j.juro.2016.10.071 [published Online First: 2016/12/26]

9. National Comprehensive Cancer Network. NCCN Clinical Practice Guidelines in Oncology (NCCN Guideline). Prostate Cancer., 2020.

For peer review only - http://bmjopen.bmj.com/site/about/guidelines.xhtml

10. Kluger J, Roy A, Chao HH. Androgen Deprivation Therapy and Cognitive Function in Prostate Cancer. *Current oncology reports* 2020;22(3):24. doi: 10.1007/s11912-020-0884-1 [published Online First: 2020/02/13]

11. Sun M, Cole AP, Hanna N, et al. Cognitive Impairment in Men with Prostate Cancer Treated with Androgen Deprivation Therapy: A Systematic Review and Meta-Analysis. *Journal of Urology* 2018;199(6):1417-25. doi: doi:10.1016/j.juro.2017.11.136

12. McHugh DJ, Root JC, Nelson CJ, et al. Androgen-deprivation therapy, dementia, and cognitive dysfunction in men with prostate cancer: How much smoke and how much fire? *Cancer* 2018;124(7):1326-34.

13. Höfer P, Lanzenberger R, Kasper S. Testosterone in the brain: Neuroimaging findings and the potential role for neuropsychopharmacology. *European Neuropsychopharmacology* 2013;23(2):79-88. doi: https://doi.org/10.1016/j.euroneuro.2012.04.013

14. Bosco C, Crawley D, Adolfsson J, et al. Quantifying the evidence for the risk of metabolic syndrome and its components following androgen deprivation therapy for prostate cancer: a meta-analysis. *PloS one* 2015;10(3):e0117344. doi: 10.1371/journal.pone.0117344 [published Online First: 2015/03/21]

15. Timilshina N, Hussain S, Breunis H, et al. Predictors of hemoglobin decline in non-metastatic prostate cancer patients on androgen deprivation therapy: a matched cohort study. *Supportive care in cancer : official journal of the Multinational Association of Supportive Care in Cancer* 2011;19(11):1815-21. doi: 10.1007/s00520-010-1023-6 [published Online First: 2010/10/19]

16. Kim B, Feldman EL. Insulin resistance as a key link for the increased risk of cognitive impairment in the metabolic syndrome. *Experimental & molecular medicine* 2015;47:e149. doi: 10.1038/emm.2015.3 [published Online First: 2015/03/15]

17. Yaffe K. Metabolic syndrome and cognitive decline. *Current Alzheimer research* 2007;4(2):123-6. [published Online First: 2007/04/14]

18. Trevisan C, Veronese N, Bolzetta F, et al. Low hemoglobin levels and the onset of cognitive impairment in older people: the PRO.V.A. Study. *Rejuvenation research* 2016;19(6):447-55. doi: 10.1089/rej.2015.1768 [published Online First: 2016/01/19]

19. Faux NG, Rembach A, Wiley J, et al. An anemia of Alzheimer's disease. *Molecular psychiatry* 2014;19(11):1227-34. doi: 10.1038/mp.2013.178 [published Online First: 2014/01/15]

For peer review only - http://bmjopen.bmj.com/site/about/guidelines.xhtml

20. Amin MB, Edge SB. AJCC cancer staging manual: springer 2017.

21. Zigmond AS, Snaith RP. The Hospital Anxiety and Depression Scale. *Acta psychiatrica Scandinavica* 1983;67(6):361-70. [published Online First: 1983/06/01]

22. Pais-Ribeiro J, Silva I, Ferreira T, et al. Validation study of a Portuguese version of the Hospital Anxiety and Depression Scale. *Psychology, health & medicine* 2007;12(2):225-35; quiz 35-7. doi: 10.1080/13548500500524088 [published Online First: 2007/03/17]

23. Buysse DJ, Reynolds CF, 3rd, Monk TH, et al. The Pittsburgh Sleep Quality Index: a new instrument for psychiatric practice and research. *Psychiatry research* 1989;28(2):193-213. doi: 10.1016/0165-1781(89)90047-4 [published Online First: 1989/05/01]

24. Marques D, Gomes AA, Meiavia A, et al. Reliability and initial validation of the Pittsburgh Sleep Quality Index, European Portuguese version: a preliminary study in a sample of higher education students. *Sleep Medicine* 2013;14:e140. doi: https://doi.org/10.1016/j.sleep.2013.11.316

25. Aaronson NK, Ahmedzai S, Bergman B, et al. The European Organization for Research and Treatment of Cancer QLQ-C30: a quality-of-life instrument for use in international clinical trials in oncology. *Journal of the National Cancer Institute* 1993;85(5):365-76. doi: 10.1093/jnci/85.5.365 [published Online First: 1993/03/03]

26. Pais-Ribeiro J, Pinto C, Santos C. Validation study of the Portuguese version of the QLC-C30-V.3. *Psicologia, Saúde & Doenças* 2008;9:89-102.

27. van Andel G, Bottomley A, Fossa SD, et al. An international field study of the EORTC QLQ-PR25: a questionnaire for assessing the health-related quality of life of patients with prostate cancer. *European journal of cancer (Oxford, England : 1990)* 2008;44(16):2418-24. doi: 10.1016/j.ejca.2008.07.030 [published Online First: 2008/09/09]

28. Herdman M, Gudex C, Lloyd A, et al. Development and preliminary testing of the new five-level version of EQ-5D (EQ-5D-5L). *Quality of life research : an international journal of quality of life aspects of treatment, care and rehabilitation* 2011;20(10):1727-36. doi: 10.1007/s11136-011-9903-x [published Online First: 2011/04/12]

29. Rawson KA, Gunstad J, Hughes J, et al. The METER: a brief, self-administered measure of health literacy. *Journal of general internal medicine* 2010;25(1):67-71. doi: 10.1007/s11606-009-1158-7 [published Online First: 2009/11/04]

For peer review only - http://bmjopen.bmj.com/site/about/guidelines.xhtml

30. Paiva D, Silva S, Severo M, et al. Cross-cultural adaptation and validation of the health literacy assessment tool METER in the Portuguese adult population. *Patient education and counseling* 2014;97(2):269-75. doi: 10.1016/j.pec.2014.07.024 [published Online First: 2014/08/12]

31. Nasreddine ZS, Phillips NA, Bedirian V, et al. The Montreal Cognitive Assessment, MoCA: a brief screening tool for mild cognitive impairment. *Journal of the American Geriatrics Society* 2005;53(4):695-9. doi: 10.1111/j.1532-5415.2005.53221.x [published Online First: 2005/04/09]

32. Freitas S, Simoes MR, Alves L, et al. Montreal Cognitive Assessment (MoCA): normative study for the Portuguese population. *Journal of clinical and experimental neuropsychology* 2011;33(9):989-96. doi: 10.1080/13803395.2011.589374 [published Online First: 2011/11/16]

33. Pinto P, Severo M, Ruano L, et al. The Cube Test: a novel cognitive screening tool. *Gaceta Sanitaria* 2018;Suppl. 32:108.

34. Ruano L, Sousa A, Severo M, et al. Development of a self-administered web-based test for longitudinal cognitive assessment. *Scientific reports* 2016;6:19114. doi: 10.1038/srep19114 [published Online First: 2016/01/09]

35. Freitas S, Simões MR, Martins C, et al. Estudos de adaptação do Montreal Cognitive Assessment (MoCA) para a população Portuguesa. *Avaliação Psicológica* 2010;9:345-57.

36. Ruano L, Severo M, Sousa A, et al. Tracking cognitive performance in the general population and in patients with mild cognitive impairment with a self-applied computerized test (Brain on Track). *Journal of Alzheimer's Disease* 2019;71(2):541-48.

37. Winblad B, Palmer K, Kivipelto M, et al. Mild cognitive impairment--beyond controversies, towards a consensus: report of the International Working Group on Mild Cognitive Impairment. *Journal of internal medicine* 2004;256(3):240-6. doi: 10.1111/j.1365-2796.2004.01380.x [published Online First: 2004/08/25]

38. American Psychiatric Association. Major neurocognitive disorder. Diagnostic and Statistical Manual of Mental Disorders, Fifth Edition (DSM-5). Arlington, VA: American Psychiatric Association 2013:602-603.

39. Morris JC. The Clinical Dementia Rating (CDR): current version and scoring rules. *Neurology* 1993;43(11):2412-4. [published Online First: 1993/11/01]

40. Kalbfleisch J, Prentice R. The statistical analysis of failure time data. 2nd ed. New York, NY: John Wiley & Sons 2002:203-215.

For peer review only

41. Ruano L, Araújo N, Branco M, et al. Prevalence and Causes of Cognitive Impairment and Dementia in a Population-Based Cohort From Northern Portugal. *American Journal of Alzheimer's Disease & Other Dementiasr* 2018;34(1):49-56. doi: 10.1177/1533317518813550

42. Pereira S, Fontes F, Sonin T, et al. Neurological complications of breast cancer: study protocol of a prospective cohort study. *BMJ open* 2014;4(10):e006301. doi: 10.1136/bmjopen-2014-006301 [published Online First: 2014/10/30]

43. RORENO. Sobrevivência global, Doentes diagnosticados em 2009-10 Região Norte. Porto, Portugal 2017.

44. Monteiro I, Morais S, Costa AR, et al. Changes in employment status up to 5 years after breast cancer diagnosis: A prospective cohort study. *Breast* 2019;48:38-44. doi: 10.1016/j.breast.2019.07.007 [published Online First: 2019/09/08]

45. Gino S, Mendes T, Maroco J, et al. Memory complaints are frequent but qualitatively different in young and elderly healthy people. *Gerontology* 2010;56(3):272-7. doi: 10.1159/000240048 [published Online First: 2009/09/25]

46. Schmand B, Jonker C, Hooijer C, et al. Subjective memory complaints may announce dementia. *Neurology* 1996;46(1):121-5. [published Online First: 1996/01/01]

47. Borkowski JG, Benton AL, Spreen O. Word fluency and brain damage. *Neuropsychologia* 1967;5(2):135-40. doi: 10.1016/0028-3932(67)90015-2

48. Cavaco S, Goncalves A, Pinto C, et al. Semantic fluency and phonemic fluency: regression-based norms for the Portuguese population. *Archives of clinical neuropsychology : the official journal of the National Academy of Neuropsychologists* 2013;28(3):262-71. doi: 10.1093/arclin/act001 [published Online First: 2013/01/24]

49. Babins L, Slater ME, Whitehead V, et al. Can an 18-point clock-drawing scoring system predict dementia in elderly individuals with mild cognitive impairment? *Journal of clinical and experimental neuropsychology* 2008;30(2):173-86. doi: 10.1080/13803390701336411 [published Online First: 2008/10/22]

50. Santana I, Duro D, Freitas S, et al. The Clock Drawing Test: Portuguese norms, by age and education, for three different scoring systems. *Archives of clinical neuropsychology : the official journal of the*

For peer review only

National Academy of Neuropsychologists 2013;28(4):375-87. doi: 10.1093/arclin/act016 [published Online First: 2013/03/23]

51. Duro D, Freitas S, Alves L, et al. O Teste do Desenho do Relógio: influência das variáveis sociodemográficas e de saúde na população portuguesa. *Sinapse* 2012;12(1):5-12.

52. Cavaco S, Goncalves A, Pinto C, et al. Trail Making Test: regression-based norms for the Portuguese population. *Archives of clinical neuropsychology : the official journal of the National Academy of Neuropsychologists* 2013;28(2):189-98. doi: 10.1093/arclin/acs115 [published Online First: 2013/01/10]

53. Army Individual Test Battery. Manual of directions and scoring. Washington, DC: War Department, Adjutant Generals Office 1994.

54. Wechsler D. WMS-III Wechsler Memory Scale-Third Edition. San Antonio, TX: The Psychological Corporation 1997.

55. Weschler D. Escala de Memória de Weschler – 3ª Edição (WMS-III): Manual técnico. Lisboa, Portugal: CEGOC 2008.

56. Wechsler D. WAIS-III Wechsler Adult Intelligence Scale-Third Edition. San Antonio, TX: The Psychological Corporation 1997.

57. Weschler D. Escala de Inteligência de Weschler para Adultos – 3ª Edição (WAIS-III): Manual técnico. Lisboa, Portugal: CEGOC 2008.

58. Fernandes S. Stroop: Teste de Cores e Palavras. Lisboa, Portugal: CEGOC 2013.

59. Golden C. Stroop Colour and Word Test: a manual for clinical and experimental uses. Chicago, IL: Soelting 1978.

60. Guerreiro M. Contributo da neuropsicologia para o estudo das demências. Tese de doutoramento não publicada. Lisboa, Portugal: Faculdade de Medicina de Lisboa 1998.

61. Benedict RH, DeLuca J, Phillips G, et al. Validity of the Symbol Digit Modalities Test as a cognition performance outcome measure for multiple sclerosis. *Multiple sclerosis (Houndmills, Basingstoke, England)* 2017;23(5):721-33. doi: 10.1177/1352458517690821 [published Online First: 2017/02/17]

62. Alves L, Simoes MR, Martins C. The estimation of premorbid intelligence levels among Portuguese speakers: the Irregular Word Reading Test (TeLPI). *Archives of clinical neuropsychology : the official journal of the National Academy of Neuropsychologists* 2012;27(1):58-68. doi: 10.1093/arclin/acr103 [published Online First: 2011/12/06]

For peer review only

For peer review only - http://bmjopen.bmj.com/site/about/guidelines.xhtml

63. Campos RC, Gonçalves B. The Portuguese version of the Beck Depression Inventory-II (BDI-II): Preliminary psychometric data with two nonclinical samples. *European Journal of Psychological Assessment* 2011;27(4):258-64. doi: 10.1027/1015-5759/a000072

64. Beck AT, Steer RA, Brown GK. BDI-II Manual. San Antonio, TX: The Psychological Corporation 1996.

65. Mahoney FI, Barthel DW. Funcional evaluation: the Barthel Index. *Maryland state medical journal* 1965;14:61-5. [published Online First: 1965/02/01]

66. Sequeira C. Cuidar de idosos dependentes. Coimbra, Portugal: Quarteto Editora 2007.

67. Lawton MP, Brody EM. Assessment of older people: self-maintaining and instrumental activities of daily living. *The Gerontologist* 1969;9(3):179-86. [published Online First: 1969/01/01]

68. Madureira S, Moleiro C, Verdelho A, et al. Escala de Atividades Instrumentais de Vida Diária (AIVD). In: Simões MR, Santana I, Grupo de Estudos de Envelhecimento Cerebral e Demência, eds. Escalas e Testes na Demência, 3ª Edição. Lisboa, Portugal: Novartis 2015:140-45.

For peer review only

For peer review only - http://bmjopen.bmj.com/site/about/guidelines.xhtml

Table 1. Description of the instruments used for the evaluation of all patients.

Instrument	Description	Domains/subscales	Score
**MoCA**[31 35]	Test for the rapid screening of mild cognitive impairment – an intermediate clinical state between normal cognitive aging and dementia.	Attention and concentration; executive functions; memory; language; visuoconstructional skills; calculations; orientation.	Range: 0 to 30<br><br>Higher scores represent better cognitive performance.
**Cube Test**[33]	A two-task cognitive screening tool that consists in completing a 3D cube from six pieces (task 1) and remembering the position of the six pieces on a grid with 25 squares measuring eight by eight centimeters from a previously shown scheme (task 2).	Visuoconstructional skills; executive function; processing speed; delayed memory.	Time to construct the first vertex and to complete cube and the number of pieces correctly assembled in up to six minutes (task 1); number of pieces correctly positioned on the grid (task 2).
**Brain on Track**[34]	A self-administered computerized test intended for longitudinal cognitive testing that includes eight subtests.	Attention; memory; executive functions; language; calculation; constructive ability; visuospatial processing.	Range: virtually unlimited (maximum number of correct answers in a fixed time)<br><br>Higher scores represent better cognitive performance.<br><br>Scores falling below an expected performance threshold for each age/education group; a pattern of decline in individual performance.
**HADS**[21 22]	Scale with 14 questions assessing anxiety and emotional distress among patients during the previous week.	Depression; anxiety.	Range (for each subscale): 0 to 21<br><br>Scores greater than or equal to 11 represent a case of anxiety or depression, as applicable.
**PSQI**[23 24]	Index with 18 questions assessing sleep quality and disturbances during the previous month.	Subjective sleep quality; sleep latency; duration of sleep; habitual sleep efficiency; sleep disorders; use of medications for sleep; daytime dysfunction.	Range: 0 to 21<br><br>Scores greater than five indicate poor sleep quality.
**QLQ-C30**[25 26]	Scale with 30 questions assessing quality of life in patients with cancer during the previous week.	Global health status.<br><br>Functional scales: physical functioning; role functioning; emotional functioning; cognitive functioning; social functioning.<br><br>Symptom scales/items: fatigue; nausea and vomiting; pain; dyspnoea; insomnia; appetite loss; constipation; diarrhoea; financial difficulties.	Range (scales and single-item): 0 to 100<br><br>Higher scores for the global health status and for a functional scale represent a healthy level of quality of life and functioning, respectively.<br><br>Higher scores for a symptom scale/item represents a higher level of symptomatology/ problems.
**QLQ-PR25**[27]	Specific prostate cancer scale with 25 questions assessing quality of life in patients	Functional scales: sexual activity; sexual functioning.	Range (scales and single-item): 0 to 100

For peer review only - http://bmjopen.bmj.com/site/about/guidelines.xhtml

	with prostate cancer during the previous week and the last four weeks.	Symptom scales: urinary symptoms; bowel symptoms; hormonal treatment-related symptoms; incontinence aid.	Higher scores for a function scale/item reflect a healthy level of functioning.<br><br>Higher scores for a symptom scale/item reflect a higher level of symptomatology/problems.
**EQ-5D-5L**[28]	A measure of health-related quality of life with five questions and a visual analogue scale.	Mobility, self-care, usual activities, pain/discomfort; anxiety/depression; and a visual analogue scale.	A total of 3125 possible health states are defined to describe the patient's health state. Each state is referred to in terms of a 5-digit code.<br><br>Vertical visual analogue scale<br>Range: 0 to 100<br><br>Higher scores reflect 'The best health you can imagine' and lower scores reflect 'The worst health you can imagine'.
**METER**[29 30]	A measure of health literary including 40 words and 30 non-words.	40 words and 30 non-words.	Range: 0 to 40 and 0 to 30<br><br>Adequate health literacy is defined as scoring at least 35/40 in words and 18/30 in non-words.

MoCA – Montreal Cognitive Assessment; BoT – Brain on Track; HADS – Hospital Anxiety and Depression Scale; PSQI – Pittsburgh Sleep Quality Index; QLQ-C30 – Quality of Life Questionnaire of the European Organization for Research and Treatment of Cancer; QLQ-PR25 – Prostate cancer-specific module of the Quality of Life Questionnaire of the European Organization for Research and Treatment of Cancer; EQ-5D-5L – Measure of health-related quality of life of the EuroQol Group; METER, Medical Term Recognition Test.

For peer review only

For peer review only - http://bmjopen.bmj.com/site/about/guidelines.xhtml

Table 2. Description of the battery of instruments used for the neuropsychological evaluation of the subcohort of patients with suspected cognitive impairment*.

Instrument	Description	Cognitive domains/function	Score
**SMC**[45 46]	A 10-item scale regarding subjective memory complaints.	Subjective memory.	Range: 0 to 21<br><br>Higher scores reflect maximal memory complaints.
**Phonemic Verbal Fluency**[47 48]	A test consisting of three trials of one minute each where participants are asked to produce orally as many words as possible beginning with a specific letter.	Executive function; language; semantic memory.	The total trial score corresponds to the number of words correctly produced within one minute. The total test score corresponds to the sum of the three trials.<br><br>Higher scores correspond to better performance.
**18-point CDT**[49-51]	An 18-point clock-drawing scoring system where participants are asked to draw a big circle and put the numbers of the clock, and then they were asked to indicate the time as "10 past 11".	Visuospatial; executive function.	Range: 0 to 18<br><br>Scoring system with three main components: (a) assessment of circle integrity (two points); (b) number placement and sequencing (six points); and (c) placement and size of the hands (six points). Additionally, there are two points for representation of the clock's center and two points for general gestalt.
**TMT**[52 53]	Part A: participants are asked to draw lines to connect 25 randomly positioned numbered circles in numeric order as quickly as possible.<br><br>Part B: participants are asked to draw lines to connect circles in numeric and alphabetic order as quickly as possible, alternating between numbers and letters (progressively up to number 13).	Part A: attention; visual scanning; and speed of eye-hand coordination and information processing.<br><br>Part B: working memory and executive functions; particularly, the ability to switch between sets of stimuli.	Direct measures of performance: time (seconds) to complete part A and part B and performance errors during part A and part B.<br><br>Derived scores: difference score (B - A), ratio score (B/A), proportion score (B - A/A), sum score (A + B), and multiplication score (A × B/100).<br><br>Lower raw scores and higher adjusted scores correspond to better performance.
**WMS-III**[54 55]	Evaluates memory and attention functions using both auditory and visual stimuli.<br><br>A test composed of 17 subtests designed to measure different memory functions in a person with the aim of detecting and discriminating between subcortical vascular dementia and Alzheimer´s disease.	Verbal and visual memories; working memory.	Range:<br>- Immediate recall: 0-50<br>- Delayed recall: 0-50<br>- Auditory recognition: 0-30<br>- Visual reproduction: 0-104<br>- Digit span: 0-30

For peer review only - http://bmjopen.bmj.com/site/about/guidelines.xhtml

	Subtests used: Logical Memory I, II; Visual Reproduction I, II; Digit Span		Higher scores correspond to better performance.
**WAIS-III**[56 57]	Measures intelligence and cognitive ability in adults and older adolescents.<br><br>Subtests used: Digit-Symbol-Coding which consists of digit-symbol pairs followed by a list of digits and under each digit the participants write down the corresponding symbol as fast as possible; and Symbol Search, in which, the participants are asked to look at two groups of symbols and to indicate if any of the symbols of the first group are present in the other group.	Attention/concentration; executive function (sequencing); motor function; processing speed.	The number of correct symbols within the allowed time (120 seconds) is measured.
**Stroop Test**[58 59]	Assesses the ability to inhibit cognitive interference, which occurs when the processing of a stimulus feature affects the simultaneous processing of another attribute of the same stimulus.<br><br>This test has three trials: 1) the participant is required to read the colour names printed in black ink as quickly as possible; 2) the participant is required to name the colour of colored dots as quickly as possible; 3) the participant is required to name the colour of the ink of the colour name words (the colour name does not match the colour of the ink).	Executive functions (inhibitory control); selective attention.	Scores for each trial indicate the number of correct responses. An interference score can be generated that quantifies the participant's ability to inhibit the inappropriate response of reading the colour name as opposed to the colour of the ink used to print the colour name in the third trial.
**Token Test – short form**[60]	A test designed to assess the comprehension of commands that vary in degree of linguistic difficulty but which are relatively independent of defects in other aspects of intellectual capacity such as memory and vocabulary.<br><br>The test consists of six subsections which represent different levels of linguistic difficulty. The participant is presented with tokens of different shapes (i.e., circles, squares, triangles), sizes, and colours, and is required to perform certain acts with the tokens, such as point to selected tokens,	Attention and vigilance; verbal functions.	Range: 0-36

For peer review only - http://bmjopen.bmj.com/site/about/guidelines.xhtml

	touch them, pick them up, and place one token on top of another.		
**SDMT**[61]	A quick screening test for organic cerebral dysfunction.<br><br>The test involves a simple substitution task that can be easily performed: using a reference key, the participant has 90 seconds to pair specific numbers with given geometric figures.	Organic cerebral dysfunction.	Individuals with cerebral dysfunction perform poorly.
**TeLPI**[62]	A Portuguese irregular word reading test using 46 irregular, infrequent Portuguese words designed to assess premorbid intelligence.	Premorbid Intelligence Quotient (IQ): full scale IQ; Verbal IQ; performance IQ	Range: number of errors (maximum of 46) and years of education are inserted in three linear equations to calculate the three types of IQ
**BDI-II**[63 64]	A 21 question measure assessing the presence of depressive symptoms experienced by the participant within the past week.	Emotional functioning.	Range: 0 to 63<br><br>A cut-off score indicative of mild depressive symptoms is greater than 10 and for severe depressive symptoms is greater than 30.
**Barthel ADL Index**[65 66]	An index to measure functional disability, focused on bodily oriented personal care.	Functional domains: feeding; incontinence; transferring; toileting; dressing; bathing.	Range: 0 to 100<br><br>Lower scores reflect increased disability.
**IADL**[67 68]	An eight item scale used to assess independent living skills which include more complex activities (i.e. “instrumental activities of daily living”) necessary for functioning in community settings.	Functional domains: using the telephone; shopping; food preparation; housekeeping; laundry; transport; medication; finances.	Range: 0 to 8<br><br>Higher scores reflect high function, independence.

SMC – Subjective Memory Complains scale; CDT – Clock Drawing Test; TMT – Trail Making Test; WMS-III – Wechsler Memory Scale – Third Edition; WAIS-III – Wechsler Adult Intelligence Scale – Third Edition; SDMT – Symbol and Digit Modalities Test; TeLPI – Irregular Word Reading Test; BDI-II – Beck Depression Inventory – Second Edition; Barthel ADL Index – Barthel Activities of Daily Living Index; IADL – Lawton and Brody Instrumental Activities of Daily Living.

* Only patients who score below 1.5 standard deviations of age- and education-adjusted cut-offs at the Montreal Cognitive Assessment during each evaluation (baseline, and one, three, five, seven and 10 years of follow-up) will be invited for a neuropsychological evaluation.

For peer review only

For peer review only - http://bmjopen.bmj.com/site/about/guidelines.xhtml

FIGURE 1. STUDY DESIGN, AND TIMING OF BASELINE AND FOLLOW-UP EVALUATIONS IN THE MAIN COHORT AND THE SUBCOHORT OF PARTICIPANTS WITH SUSPECTED COGNITIVE IMPAIRMENT.

SD – standard deviations.

* Subsequent follow-up evaluations will be at three, five, seven and 10 years after the baseline evaluation.

† The Brain on Track evaluation will be conducted every three months for a period of two years.

‡Only participants who score below 1.5 standard deviations of age- and education-adjusted cut-offs on the Montreal Cognitive Assessment during each evaluation (baseline, and one, three, five, seven and 10 years of follow-up) will be invited for a neuropsychological evaluation where a battery of cognitive tests will be applied. The type of cognitive impairment will be classified through a clinical evaluation performed by a neurologist.

For peer review only

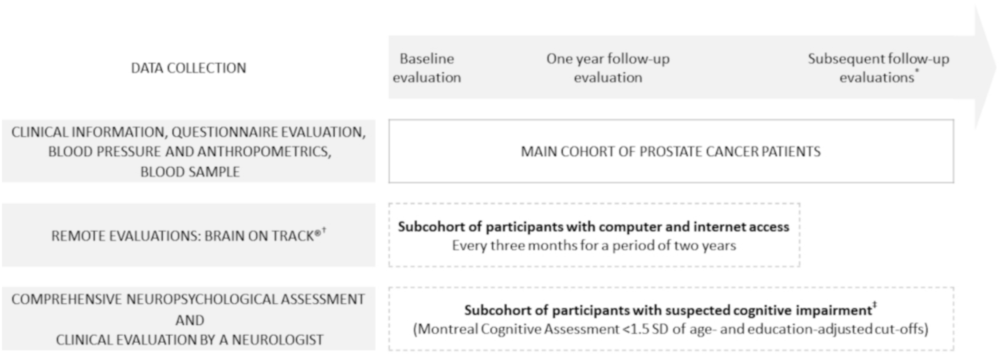

Figure 1. Study design, and timing of baseline and follow-up evaluations in the main cohort and the subcohort of participants with suspected cognitive impairment.
SD – standard deviations.
* Subsequent follow-up evaluations will be at three, five, seven and 10 years after the baseline evaluation.
† The Brain on Track evaluation will be conducted every three months for a period of two years.
‡Only participants who score below 1.5 standard deviations of age- and education-adjusted cut-offs on the Montreal Cognitive Assessment during each evaluation (baseline, and one, three, five, seven and 10 years of follow-up) will be invited for a neuropsychological evaluation where a battery of cognitive tests will be applied. The type of cognitive impairment will be classified through a clinical evaluation performed by a neurologist.

160x90mm (300 x 300 DPI)
